# Variants of the human *RAD52* gene confer defects in ionizing radiation resistance and homologous recombination repair in budding yeast

**DOI:** 10.15698/mic2020.10.732

**Published:** 2020-07-20

**Authors:** Alissa D. Clear, Glenn M. Manthey, Olivia Lewis, Isabelle Y. Lopez, Rossana Rico, Shannon Owens, M. Cristina Negritto, Elise W. Wolf, Jason Xu, Nikola Kenjić, J. Jefferson P. Perry, Aaron W. Adamson, Susan L. Neuhausen, Adam M. Bailis

**Affiliations:** 1 Department of Molecular and Cellular Biology, Beckman Research Institute of City of Hope, Duarte, CA, USA.; 2 Irell & Manella Graduate School of Biological Sciences, Beckman Research Institute of City of Hope, Duarte, CA, USA.; 3 bioStrategies Group, Chicago, IL, USA.; 4 City of Hope – Duarte High School NIH Science Education Partnership Award Program, Duarte, CA, USA.; 5 Barbara Bush Houston Literacy Foundation, Houston, TX, USA.; 6 California State Polytechnic University at Pomona, Pomona, CA, USA.; 7 Henry Samueli School of Engineering and Applied Sciences, University of California at Los Angeles, Los Angeles, CA, USA.; 8 Eugene and Ruth Roberts Summer Student Academy, Beckman Research Institute of City of Hope, Duarte, CA, USA.; 9 Department of Microbiology and Molecular Genetics, College of Biological Sciences, University of California at Davis, Davis, CA, USA.; 10 Molecular Biology Program, Pomona College, Claremont, CA, USA.; 11 Department of Microbiology and Immunology, University of California at San Francisco, San Francisco, CA, USA.; 12 Perelman School of Medicine, University of Pennsylvania, Philadelphia, PA, USA.; 13 Department of Biochemistry, University of California at Riverside, Riverside, CA, USA.; 14 Department of Population Sciences, Beckman Research Institute of City of Hope, Duarte, CA, USA.; 15 College of Health Professions, Thomas Jefferson University, Philadelphia, PA, USA.

**Keywords:** HsRAD52 variants, tumorigenesis, DNA double strand breaks, homologous recombination repair, ionizing radiation, budding yeast

## Abstract

RAD52 is a structurally and functionally conserved component of the DNA double-strand break (DSB) repair apparatus from budding yeast to humans. We recently showed that expressing the human gene, *HsRAD52* in *rad52* mutant budding yeast cells can suppress both their ionizing radiation (IR) sensitivity and homologous recombination repair (HRR) defects. Intriguingly, we observed that *HsRAD52* supports DSB repair by a mechanism of HRR that conserves genome structure and is independent of the canonical HR machinery. In this study we report that naturally occurring variants of *HsRAD52*, one of which suppresses the pathogenicity of *BRCA2* mutations, were unable to suppress the IR sensitivity and HRR defects of *rad52* mutant yeast cells, but fully suppressed a defect in DSB repair by single-strand annealing (SSA). This failure to suppress both IR sensitivity and the HRR defect correlated with an inability of HsRAD52 protein to associate with and drive an interaction between genomic sequences during DSB repair by HRR. These results suggest that HsRAD52 supports multiple, distinct DSB repair apparatuses in budding yeast cells and help further define its mechanism of action in HRR. They also imply that disruption of HsRAD52-dependent HRR in BRCA2-defective human cells may contribute to protection against tumorigenesis and provide a target for killing BRCA2-defective cancers.

## INTRODUCTION

Ionizing radiation (IR) is a ubiquitous component of our environment that arises from both natural and manmade sources. Human exposure to IR results in damage at the cellular level, with DNA damage thought to be the source of its lethal effect. IR induces a variety of physical and chemical changes to the DNA, with the double-strand break (DSB) being the most lethal [1–4]. Accordingly, biological systems have evolved multiple mechanisms for the repair of DSBs that contribute to IR resistance [5–11]. The most prominent of these mechanisms are homologous recombination repair (HRR) and non-homologous end joining (NHEJ), which are genetically and biochemically distinct. These distinctions are the basis of the different outcomes of DSB repair by HRR and NHEJ, with HRR most frequently conserving genome structure and NHEJ frequently altering it [12–14]. The balance between these conservative and non-conservative mechanisms of DSB repair can have profound effects on genome integrity and human health following exposure to IR [15].

Intriguingly, the balance between conservative HRR and non-conservative NHEJ in DSB repair favors NHEJ in human cells, perhaps reflecting the extraordinary speed and efficiency of the mechanism [16]. However, mutations in several HRR genes, including *ATM, MRE11*, and *XRCC3* can increase sensitivity to IR and cancer susceptibility [17], indicating the relevance of DSB repair by HRR in the response of human cells to IR exposure. An interesting facet of DSB repair by HRR was revealed when attenuation of the HR factor, RAD52, in addition to canonical HRR pathway defects had profoundly synergistic effects on DSB repair by HRR, suggesting that it may support a distinct mechanism of HRR [18–21]. Independent localization of RAD52 and canonical HRR factors to IR-induced DNA damage confirmed their distinct cellular response to DSBs and the probable existence of separate repair apparatuses [22]. The synthetic lethality observed upon simultaneous attenuation of RAD52 and various canonical HRR factors, particularly BRCA1 and BRCA2 points to potential therapeutic opportunities presented by disrupting the activities of both apparatuses [18–21, 23–28], which may include sensitization of tumors to therapeutic IR.

Advancing the development of effective inhibitors of RAD52 would benefit from a more complete understanding of its function at the molecular level during DSB repair in living cells. Studying the function of human RAD52 (hereafter referred to as HsRAD52) in IR resistance and HRR in budding yeast cells has provided a compelling model as our previous investigation showed that expressing the *HsRAD52* gene in *rad52* mutant yeast strains suppresses both their IR sensitivity and HRR defects [29]. Further, *HsRAD52*-dependent HRR and association of HsRAD52 with DSBs during repair are independent of the central HR factor, Rad51 and other members of the canonical HRR apparatus of yeast, paralleling observations in mammalian cells. In the current study we have used naturally occurring variants of *HsRAD52* identified in African American women with breast cancer, *HsRAD52-G59R* and *HsRAD52-S346X* to further study the control of *HsRAD52*-dependent HRR at the genetic and molecular levels in budding yeast. We found that neither allele suppressed the IR sensitivity or defective DSB repair by conservative HRR of *rad52* mutant cells. Our recent observation that *HsRAD52-S346X* protects against breast cancer in carriers of pathogenic *BRCA2* mutations [30] suggests the possibility that a similar loss of HsRAD52-dependent HRR in human cells may contribute to the synthetic lethality that underlies this protection. Together, these observations further demonstrate the utility of the yeast model system for studying the function of HsRAD52 at the DNA level, and highlight a potential avenue for exploring the mechanisms by which inhibitors block DSB repair in living cells.

## RESULTS

### Expression of the *HsRAD52-G59R* and *HsRAD52-S346X* variant alleles in budding yeast cells produce stable proteins

**Figure 1:**
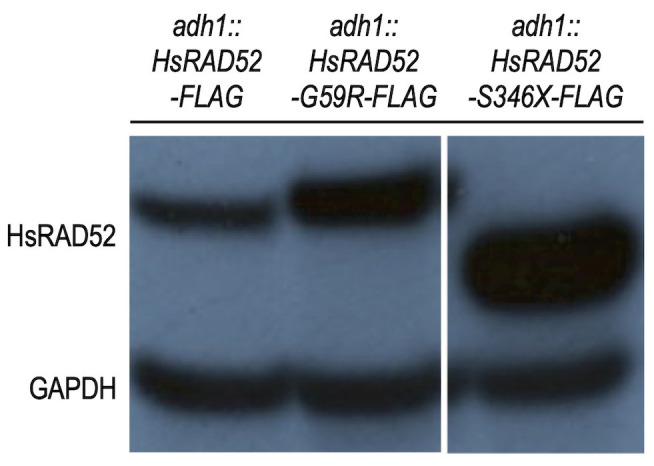
FIGURE 1: The *adh1::HsRAD52-G59R-FLAG* and *adh1::HsRAD52-S346X-FLAG* alleles express stable proteins in budding yeast cells. Whole cell extracts from strains ABX3684-12B (*adh1::HsRAD52-FLAG*), ABX3782-2D (*adh1::HsRAD52-G59R-FLAG*) and ABX3974-11C (*adh1::HsRAD52-S346X-FLAG*) were run on SDS-PAGE gels, blotted to a nylon membrane and probed with anti-FLAG and anti-GAPDH antibodies. The genotypes of the strains are denoted at the top of the figure. Bands corresponding to wild-type and mutant HsRAD52, and GAPDH are labeled on the left side of the figure.

As HsRAD52 plays a role in DSB repair by HRR [19–21] and defects in HRR are linked to cancer susceptibility [31–34], we reasoned that variants of *HsRAD52* from patients with cancer might confer loss of protein function. Accordingly, we examined the effects of *HsRAD52-G59R* and *HsRAD52-S346X,* two variants identified in a screen of African-American women with breast cancer, for their effects on the function of HsRAD52 in budding yeast cells. Utilizing our previous strategy for obtaining robust expression of *HsRAD52* in yeast [29], we inserted cDNAs of the *HsRAD52-G59R* and *HsRAD52-S346X* alleles into the *ADH1* locus such that their expression was controlled by the *ADH1* promoter and terminator sequences. Strains expressing C-terminally FLAG-tagged recombinants also were produced in order to facilitate immunological detection and selection of the proteins. As observed previously with strains expressing the *adh1::HsRAD52-FLAG* allele [29], Western blots of whole cell extracts from strains expressing the *adh1::HsRAD52-G59R-FLAG* allele produced a single, stable 49 kDa protein, with the steady-state level of the mutant protein appearing to exceed that of wild-type (**Figure 1**). Strains expressing the *adh1::HsRAD52-S346X-FLAG* allele displayed a single 38 kDa protein consistent with the deletion of 72 amino acids from the C-terminus of HsRAD52. The truncated mutant protein accumulated to a significantly higher level than either of the full-length proteins.

### The *HsRAD52-G59R* and *HsRAD52-S346X* alleles do not suppress the IR sensitivity of *rad52* mutant yeast cells

**Figure 2:**
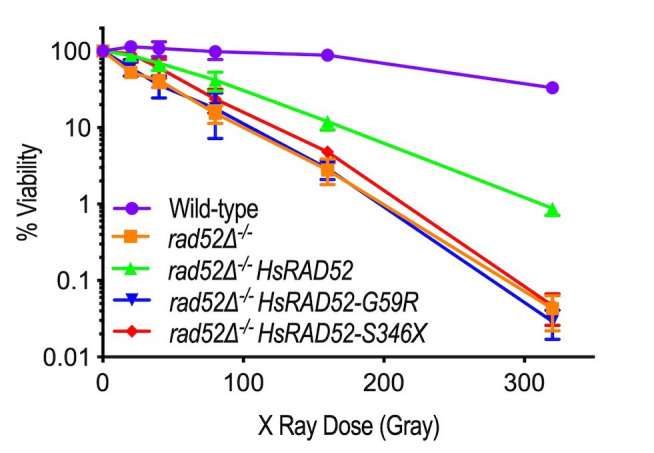
FIGURE 2: The *adh1::HsRAD52-G59R-FLAG* and *adh1::HsRAD52-S346X-FLAG* alleles fail to suppress the ionizing radiation sensitivity of *rad52*^*-/-*^ mutant yeast cells. Cultures of wild-type (ABX3566), *rad52*^*-/-*^ (ABX3568), *rad52*^*-/-*^ *adh1::HsRAD52-FLAG*^*+/+*^ (ABX4130), *rad52*^*-/-*^ *adh1::HsRAD52-G59R-FLAG*^*-/-*^ (ABX4129) and *rad52*^*-/-*^ *adh1::HsRAD52-S346X-FLAG*^*-/-*^ (ABX4131) yeast strains grown to mid-log phase were counted before being subjected to 20, 40, 80, 160 and 320 Gy of X-ray radiation. Appropriate dilutions of unirradiated and irradiated cultures were plated onto solid YPD medium, incubated at 30°C for three days, and the resulting colonies counted. Percent viability was calculated by dividing the number of colonies arising on the plates by the number of cell bodies plated and multiplying by 100. Mean percent survival was calculated for at least 10 independent cultures for each genotype. These values and 95% confidence intervals were plotted against levels of radiation exposure.

In budding yeast cells Rad52 (hereafter referred to as ScRad52) is the central factor controlling HRR, playing a predominant role in maintaining IR resistance as indicated by the exquisite sensitivity to gamma and X-ray radiation displayed by *rad52* mutant strains [9, 35, 36]. Consistent with these and our own results using a gamma radiation source [29], we observed a 767-fold decrease in viability of a *rad52*^*-/-*^ homozygous mutant diploid strain after exposure to 320 Gy of X-ray radiation relative to a wild-type diploid (**Figure 2**; Table S4). Importantly, expression of *adh1::HsRAD52-FLAG* in a *rad52*^*-/-*^ mutant diploid suppressed its sensitivity to 320 Gy of X-ray radiation by greater than 20-fold. This indicates that HsRAD52-FLAG can participate in the repair of X-ray-induced DSBs in budding yeast cells.

Having established that the *adh1::HsRAD52-G59R-FLAG* and *adh1::HsRAD52-S346X-FLAG* alleles express stable proteins at steady-state levels that are at least equal to that expressed by *adh1::HsRAD52-FLAG* (**Figure 1**), we examined their ability to suppress the IR sensitivity of the *rad52*^*-/-*^ mutant diploid. In contrast to expression of the *adh1:: HsRAD52-FLAG* allele, we observed that expression of the *adh1::HsRAD52-G59R-FLAG* and *adh1::HsRAD52-S346X-FLAG* mutant alleles in *rad52*^*-/-*^ mutant diploid strains failed to significantly suppress IR sensitivity at any X-ray dose (**Figure 2**; Table S4; p > 0.76). This indicates that the mutant HsRAD52-G59R-FLAG and HsRAD52-S346X-FLAG proteins do not function in the repair of IR-induced DSBs in budding yeast.

### The *HsRAD52-G59R* and *HsRAD52-S346X* alleles cannot suppress the HRR defect of *rad52* mutant cells

**Figure 3:**
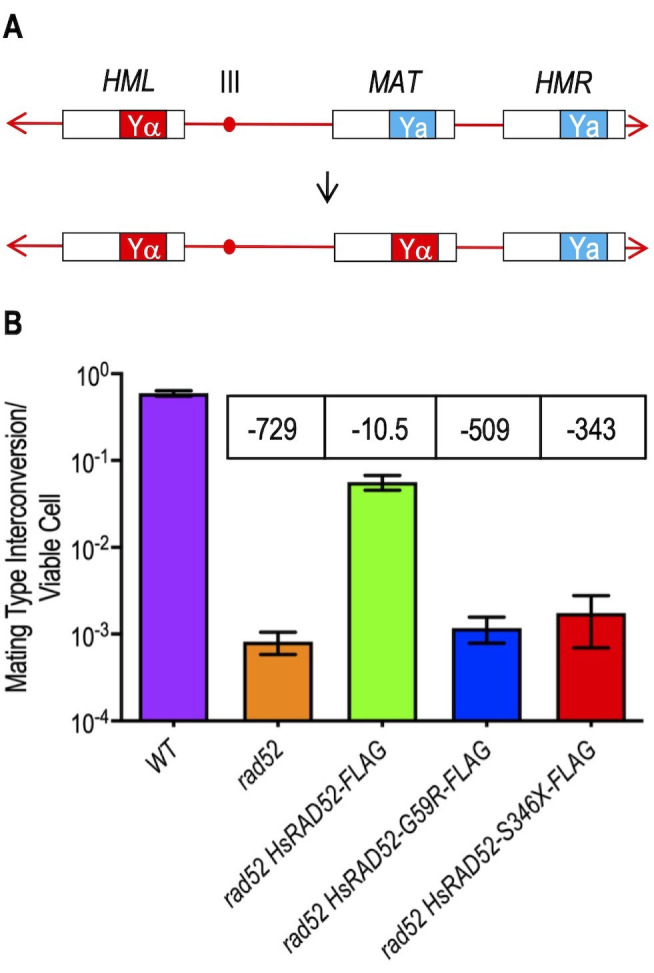
FIGURE 3: The *adh1::HsRAD52-G59R-FLAG* and *adh1::HsRAD52-S346X-FLAG* alleles do not suppress the MTI defect of *rad52* mutant yeast cells. **(A)** Cartoon depicting MTI. DSB formation by HO endonuclease cutting at the HO cut site on the centromere distal edge of the Ya sequence (blue box) at the *MAT* locus on chromosome III precipitates the exonucleolytic removal of “a” mating type information prior to its replacement by unidirectional transfer of “α” mating type information from the Yα sequence (red box) of the flanking, intact but silent *HML* locus. This results in the switching of the cell from the “a” mating type to the “α” mating type. **(B)** The *adh1::HsRAD52-G59R-FLAG* and *adh1::HsRAD52-S346X-FLAG* alleles confer defects in MTI. Single colonies of haploid wild-type (ABX3817-15B), *rad52* (ABX3817-7D), *rad52 adh1::HsRAD52-FLAG* (ABX3977-10C), *rad52 adh1::HsRAD52-G59R-FLAG* (ABX3985-55B), and *rad52 adh1::HsRAD52-S346X* (ABX3994-18D) strains were used to inoculate at least 10 one milliliter YPGL cultures and grown overnight. Following a period of expression of HO endonuclease, appropriate dilutions were plated onto YPD medium, incubated for three days at 30°, and the number of colonies counted. Colonies were replica plated to fresh YPD plates, printed with a lawn of the haploid R113a mating type tester strain, printed plates incubated overnight at 30°C and then replica plated onto SD plates, which were incubated overnight at 30°C. Frequencies of MTI were determined by dividing the number of diploid patches arising on the SD plates by the number of colonies counted on the original YPD plates. Mean frequencies of MTI and 95% confidence intervals were plotted against genotype. Fold differences below (-) the wild-type frequency of MTI for each strain are indicated in boxes above the bar for each mean frequency.

Budding yeast cells undergo mating type interconversion (MTI) by a highly efficient, programmed gene conversion event that proceeds by HRR of an HO endonuclease catalyzed DSB at the *MAT* locus on chromosome III using donor information from the flanking *HML* and *HMR* loci (**Figure 3A**) [37–39]. In accordance with its role as the central HRR factor in budding yeast, mutations in *RAD52* confer a dramatic loss in the efficiency of MTI [40]. Using a new assay for determining the frequency of MTI subsequent to galactose-induced expression of HO endonuclease, we observed a frequency near unity (59.6%) in wild-type cells that was reduced 729-fold in *rad52* mutant cells (**Figure 3B**; Table S4). Expression of *adh1::HsRAD52-FLAG* in *rad52* mutant cells suppressed the MTI defect by 69-fold indicating that HsRAD52-FLAG possesses substantial functionality in this heterologous system (**Figure 3B**; Table S4). Importantly, *adh1::HsRAD52-FLAG* also suppressed the inability of *rad52* mutant cells to repair a DSB at the *HIS3* locus by gene conversion using an unlinked, defective copy of the *HIS3* gene (Figure S1, Table S4) [29]. This indicates that HsRAD52-FLAG can support the repair of HO-catalyzed DSBs by HRR in multiple genomic contexts in budding yeast.

In contrast to expression of the *adh1::HsRAD52-FLAG gene*, we found that expression of the *adh1::HsRAD52-G59R-FLAG* (p = 0.30) and *adh1::HsRAD52-S346X-FLAG* (p = 0.31) mutant alleles failed to yield statistically significant changes in the frequencies of MTI in *rad52* mutant strains (**Figure 3B**; Table S4). We also observed that these alleles conferred similar defects in the repair of a DSB at the *HIS3* locus by HRR (Figure S1; Table S4). Combined with our observations that these alleles failed to suppress the IR sensitivity of *rad52*^*-/-*^ diploids (Table S4), these data indicate that the mutant HsRAD52-G59R-FLAG and HsRAD52-S346X-FLAG proteins do not support the repair of HO-catalyzed or IR-induced DSBs by an HR mechanism that conserves genome structure.

### Wild-type and mutant alleles of *HsRAD52* complement the loss of DSB repair by single-strand annealing (SSA) in *rad52* mutant cells

**Figure 4:**
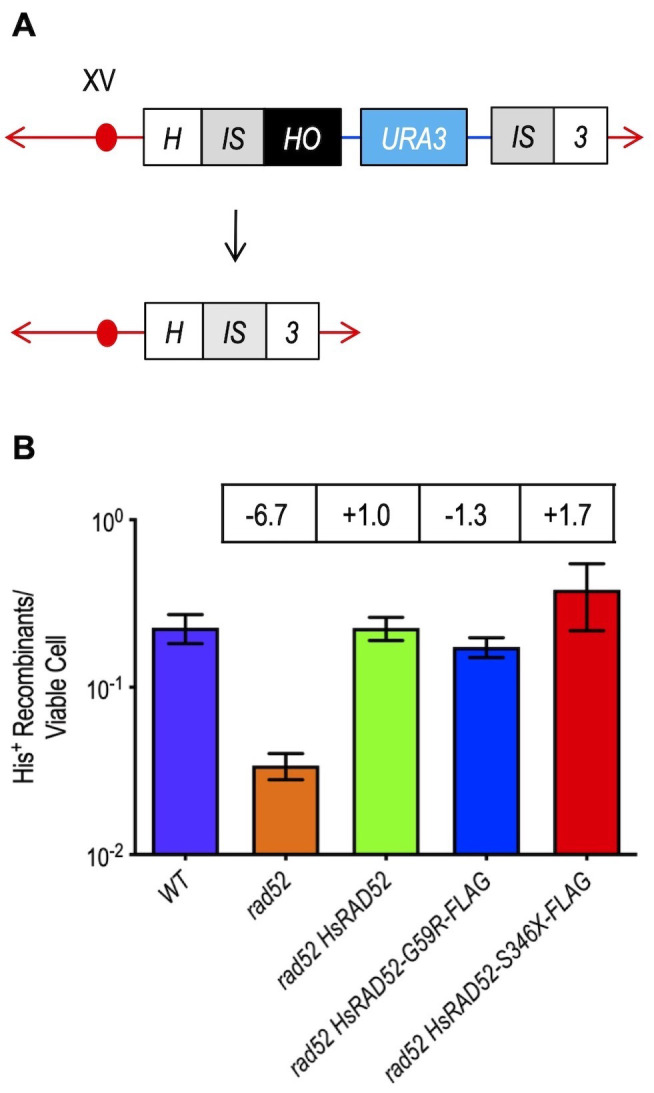
FIGURE 4: The *adh1::HsRAD52-G59R-FLAG* and *adh1::HsRAD52-S346X-FLAG* alleles complement the loss of DSB repair by SSA in *rad52* mutant yeast cells. **(A)** Cartoon depicting DSB repair by recombination between non-tandem direct repeats. At the *HIS3* locus on chromosome XV, DSB formation by HO endonuclease cutting at a HO cut site (black box) inserted at the right edge of the left duplication of a segment of the *HIS3* coding sequence (left gray *IS* box) initiates bidirectional exonucleolytic processing. Processing reveals complementary single-stranded sequences at the left and right repeats (left and right gray *IS* boxes) that anneal, creating non-homologous tails whose removal deletes intervening plasmid sequences (blue line and aqua *URA3* marker box) enroute to creating an intact *HIS3* gene. **(B)** The *adh1::HsRAD52-G59R-FLAG* and *adh1::HsRAD52-S346X-FLAG* alleles complement the defects in DSB repair by DRR. Single colonies of haploid wild-type (ABM325), *rad52* (ABM326), *rad52 adh1::HsRAD52* (ABM507), *rad52 adh1::HsRAD52-G59R-FLAG* (ABX3970-88A), and *rad52 adh1::HsRAD52-S346X* (ABX3975-15A) strains were used to inoculate at least 10 one milliliter YPGL cultures and grown overnight. After a period of expression of HO endonuclease, appropriate dilutions were plated onto solid YPD medium to determine viability, and onto medium lacking histidine to select for recombinants. Following incubation for three days at 30° colonies were counted and frequencies of DRR determined by dividing the number of His^+^ recombinants by the number of viable cells plated. Mean frequencies of DRR and 95% confidence intervals were plotted against genotype. Fold differences above (+) and below (-) wild-type are indicated in the boxes above the bar for each mean frequency.

The yeast and mammalian RAD52 proteins have been implicated in the repair of DSBs by SSA [41, 42], a non-conservative mechanism of repair that is genetically distinct from HRR [42–44]. Since the capacity of ScRad52 to propagate SSA has been localized to its N-terminus [44, 45], the region of shared amino acid sequence homology with HsRAD52 (Figure S2) [46], we examined the ability of HsRAD52 to suppress the defect in DSB repair by SSA conferred by the *rad52* mutation. We observed that repair of a HO endonuclease-catalyzed DSB at the *HIS3* locus by recombination between duplicate 415 bp segments resulting in an intact *HIS3* gene and deletion of the intervening 5 kb plasmid sequence was very efficient in wild-type cells, occurring in nearly 23% of survivors (**Figure 4**; Table S4). The frequency of DSB-DRR (direct repeat recombination) was reduced by nearly seven-fold in a *rad52* mutant strain, confirming that DSB repair by SSA in budding yeast is largely dependent on ScRad52. Surprisingly, expression of *adh1::HsRAD52-FLAG* in a *rad52* mutant strain completely suppressed this SSA defect, as frequencies of DSB-DRR were not statistically different from those observed in wild-type cells (p = 0.98). Further, the nearly 20-fold reduced rate of spontaneous recombination between the 415 bp direct repeats in *rad52* mutant cells was also completely suppressed by the expression of *adh1::HsRAD52-FLAG* (Figure S3; Table S4). These results indicate that HsRAD52-FLAG can fully replace ScRad52 for DSB-stimulated and spontaneous SSA in budding yeast cells.

In marked contrast to their effects on DSB repair by conservative HRR, expression of the *adh1::HsRAD52-G59R-FLAG* and *adh1::HsRAD52-S346X-FLAG* mutant alleles in *rad52* mutant strains completely suppressed their defects in DSB repair by non-conservative SSA (**Figure 4**; Table S4), as frequencies of DSB-DRR were not statistically different from those of wild-type (p ≥ 0.17), or *rad52 adh1::HsRAD52-FLAG* (p ≥ 0.08) strains. These data indicate that the mutant HsRAD52-G59R-FLAG and HsRAD52-S346X-FLAG proteins possess an essentially equivalent capacity to propagate DSB-stimulated SSA as ScRad52 or HsRAD52. Additionally, this establishes that the contributions of *HsRAD52* to DSB repair by HRR and SSA are genetically distinct, and likely involve separate functions of the HsRAD52 protein in budding yeast cells.

### The *HsRAD52-S346X* allele supports formation of various HsRAD52 multimers

Monomers of HsRAD52 can self-associate and form a homomeric ring structure that is thought to be of significance to its biochemical and cellular functions [47–50]. Having observed that *adh1::HsRAD52-G59R-FLAG* and *adh1::HsRAD52-S346X-FLAG* confer losses of function in IR resistance (**Figure 2**; Table S4) and DSB repair by HRR (**Figure 3B**; Figure S1; Table S4) in budding yeast, we investigated the ability of the HsRAD52-G59R and HsRAD52-S346X mutant proteins to associate and form multimers using the yeast two hybrid (Y2H) and gel filtration analyses. We observed levels of β-galactosidase activity in the extracts of yeast cells transformed with plasmids expressing the HsRAD52-Gal4 DNA binding domain and HsRAD52-Gal4 transcription activation domain fusion proteins (Table S2) that were substantially (80-fold) above background (Figure S4; Table S5). These results are consistent with the self-association of HsRAD52 monomers in budding yeast cells. Expression of the HsRAD52-G59R and HsRAD52-S346X mutant fusion proteins produced levels of β-galactosidase activity that were not statistically different from those observed with the wild-type HsRAD52 fusion proteins (p ≥ 0.51), indicating that HsRAD52-G59R and HsRAD52-S346X possess a capacity for self-association that is comparable to that of HsRAD52.

**Figure 5:**
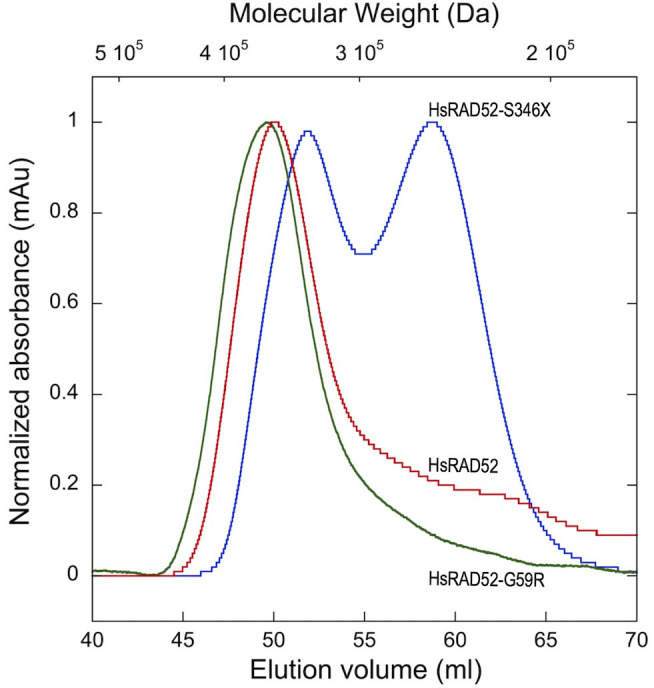
FIGURE 5: *HsRAD52-S346X* alters the in-solution oligomerization state of HsRAD52. Superdex 200 size exclusion elution profiles are shown for HsRAD52_1-212,_ HsRAD52-G59R_1-212_, and HsRAD52-S346X. HsRAD52_1-212_ (red trace) eluted as a single peak at 50.0ml, with an estimated size of 360kDa. HsRAD52-G59R_1-212_ (green trace) eluted as a single peak at 49.6 ml with an estimated size of 366kDa. HsRAD52-S346X (blue trace) eluted as two peaks; the first peak eluted at 51.9 ml with an estimated size of 335kDa, and the second peak eluted at 58.7ml with an estimated size of 257kDa. (mAU = milliabsorbance unit).

While pair-wise interactions between HsRAD52 monomers are very likely to contribute to the formation of homo-heptameric HsRAD52 ring structures, they may not be the sole determinants governing the formation and stabilization of these structures. In order to define the relative capacities of wild-type and mutant HsRAD52 proteins to form higher order multimers, we performed gel filtration chromatography to define their hydrodynamic properties. We utilized peptides containing the N-terminal 212 amino acids (1-212) of HsRAD52 and HsRAD52-G59R for these analyses, as HsRAD52_1-212_ contains the domain required for self-association [48], forms undecameric rings *in vitro* [51, 52], and is substantially more stable than the full-length protein. The HsRAD52_1-212_ and HsRAD52-G59R_1-212_ multimers displayed virtually identical column elution profiles, with peaks centered at an estimated molecular weight of 360 kDa, which could approximate a mass equivalent to thirteen to fourteen subunits of the 27 kDa HsRAD52_(1-212)_ monomers (**Figure 5**). These results suggest that the *HsRAD52-G59R* mutation does not confer marked changes in the quaternary structure of HsRAD52. In contrast, HsRAD52-S346X displayed an elution profile consistent with two multimer of relatively equal abundance; one with an estimated molecular weight of 257 kDa that approximates to a hexameric multimer of 41 kDa monomers, and a second with an estimated molecular weight of 335 kDa that would be suggestive of an octamer.

### The *HsRAD52-G59R* and *HsRAD52-S346X* alleles alter interactions between HsRAD52 and recombination substrates during DSB repair by HRR

**Figure 6:**
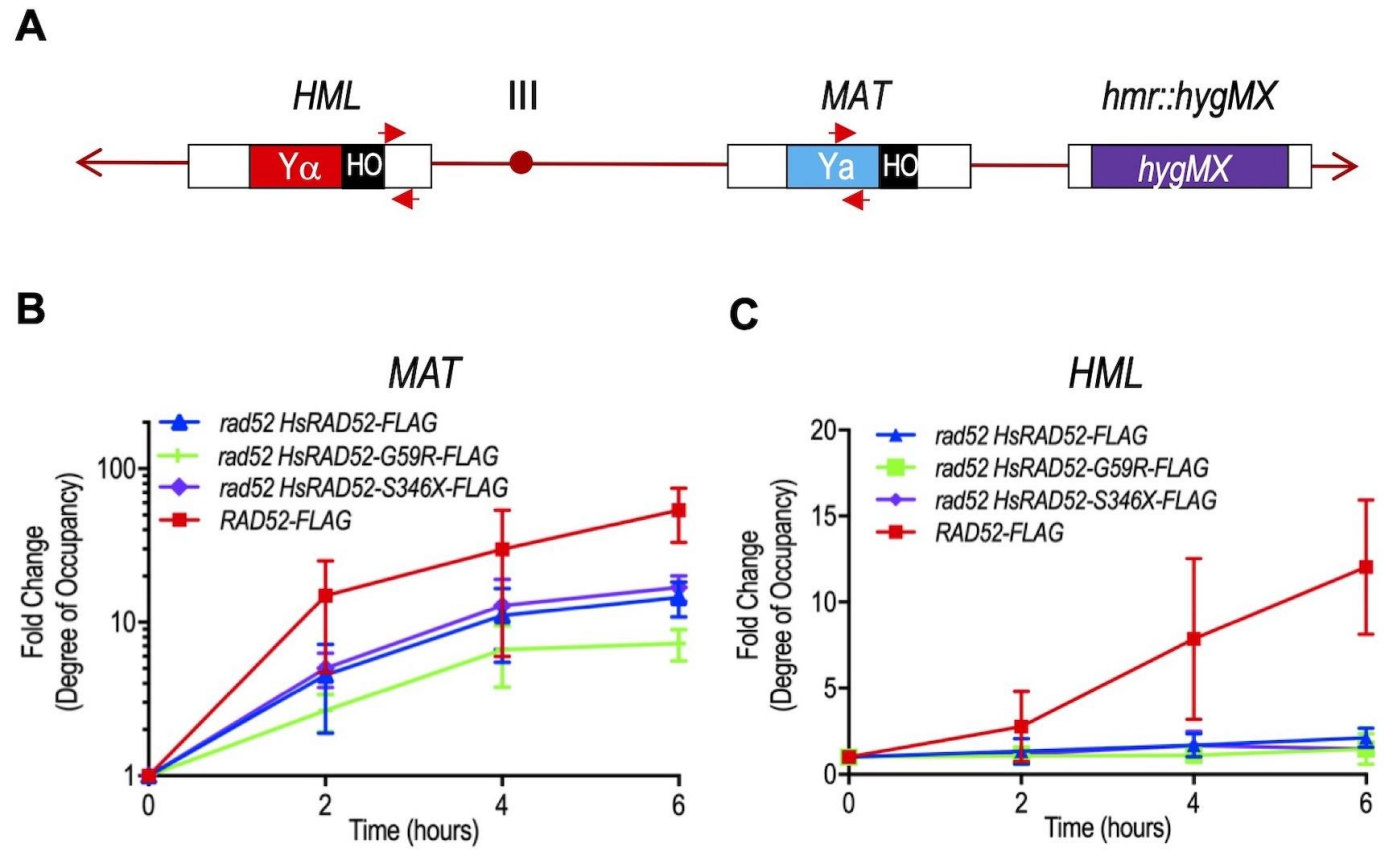
FIGURE 6: The *adh1::HsRAD52-G59R-FLAG* and *adh1::HsRAD52-S346X-FLAG* alleles have differential effects on the interaction of HsRAD52 with the *MAT* and *HML* loci during MTI. **(A)** Cartoon depicting substrates for MTI and location of primers used for quantitation of immunoprecipitated genomic sequences. Following DSB formation by HO endonuclease at the HO cut site (black box) at the *MAT* locus on chromosome III, exonucleolytic processing results in the accumulation of ssDNA in flanking sequences. ScRad52-FLAG, or HsRAD52-FLAG progressively associate with the ssDNA. This association facilitates retention of the DNA sequences by ChIP, which are quantitated by qPCR using *MAT****a*** recipient primers (red arrows; Table S3) complementary to a 110 bp sequence laying 500 bp upstream from the DSB at *MAT*. Deletion of the *HMR* locus and replacement with a *hygMX* marker (purple box) removes additional complementary sequences from the genome. After association with ssDNA at *MAT*, the HRR apparatus facilitates a search for homologous genomic sequences that gives rise to heteroduplex formation with the intact *HML* locus. The sequences that lay proximal to the HO cut site at the border of Yα (red box) are the putative initial location for heteroduplex formation. Association of ScRad52-FLAG or HsRAD52-FLAG with the heteroduplex results in retention of these sequences by ChIP, and their quantitation is done by qPCR using *HMLα* donor primers (red arrows; Table S3) complementary to a 187 bp sequence laying 67 bp downstream from the HO cut site sequence at *HML*. **(B)** ScRad52-FLAG and HsRAD52-FLAG display similar kinetics of association with sequences at the *MAT* locus after DSB formation. Single colonies of wild-type (ABX3961-4C), *rad52* (ABX3943-3B), *rad52 adh1::HsRAD52-FLAG* (ABX3977-10C), *rad52 adh1::HsRAD52-G59R-FLAG* (ABX3985-55B), and *rad52 adh1::HsRAD52-S346X-FLAG* (ABX3994-18D) strains carrying MTI assay components were used to establish cultures from which aliquots were collected at various times before and after DSB formation at the *MAT* locus by HO endonuclease. Whole cell extracts were prepared, subjected to ChIP using anti-FLAG antibody and the immunoprecipitated DNA from the *MAT* (experimental) and *SAM1* (control) loci quantitated by qPCR. Fold changes in degree of occupancy of the FLAG-tagged proteins relative to those observed before DSB formation were normalized to a control strain lacking FLAG-tagged proteins (ABX3933-46C). Mean fold changes from at least eight determinations using DNA collected from at least three independent time courses, and standard deviations were plotted against elapsed time after initiation of DSB formation. **(C)** HsRAD52-G59R-FLAG and HsRAD52-S346X-FLAG display defects in association with the *HMR* locus during MTI. Same as above except immunoprecipitated DNA from the *HMR* locus (experimental) was quantitated by qPCR.

The function of HsRAD52 in DSB repair by HRR in budding yeast cells was previously correlated with its progressive accumulation at the genomic DSB as documented by ChIP [29]. Accordingly, we used ChIP to study the association of HsRAD52-FLAG with the *MAT****a*** locus during MTI in strains where the *HMR* locus was deleted to prevent contamination by signal acquired from the “a” information at that locus (**Figure 6A**). We observed that following DSB formation, HsRAD52-FLAG accumulated at the *MAT* locus in *rad52 adh1::HsRAD52-FLAG* cells, peaking at 14.6-fold enrichment at six hours (**Figure 6B**). The HsRAD52-S346X-FLAG mutant protein associated with the *MAT* locus with kinetics and at levels that were very similar to those of HsRAD52-FLAG (p = 0.22), whereas the HsRAD52-G59R-FLAG mutant protein displayed similar kinetics, but a level of accumulation at six hours that was two-fold lower (p = 0.0002) than that of HsRAD52. Interestingly, ScRad52-FLAG also accumulated at the *MAT* locus with similar kinetics to those of HsRAD52-FLAG, but reached a nearly five-fold greater level of enrichment at six hours.

Molecular models for MTI propose that the HRR apparatus assembles at the *MAT* locus subsequent to DSB formation after which there is a search for and pairing with intact homologous sequences at the flanking *HML* or *HMR* loci [39]. Accordingly, previously published experiments indicated that ScRad52 associates sequentially with *MAT* and *HML* (or *HMR*) [53]. Our ChIP data indicated that ScRad52-FLAG associates with the *MAT* and *HML* loci with distinct kinetics. ScRad52-FLAG had accumulated 15-fold at *MAT* 2 hours after DSB formation, which was less than 4-fold below the level observed at 6 hours (**Figure 6B**). In contrast, ScRad52-FLAG had accumulated only 2.8-fold at *HML* after 2 hours before reaching a peak accumulation of 12.8-fold at 6 hours (**Figure 6C**). These data are consistent with ScRad52-FLAG partaking in separate interactions with the *MAT****a*** recipient and *HML* donor substrates during MTI in our strains.

Like ScRad52-FLAG, HsRAD52-FLAG displayed distinct kinetics of association with the *MAT* and *HML* loci subsequent to DSB formation, accumulating rapidly at *MAT* (**Figure 6B**) and slowly at *HML* (**Figure 6C**) in *rad52 adh1::HsRAD52-FLAG* strains, and consistent with HsRAD52-FLAG interacting separately with *MAT* and *HML* during MTI. In notable contrast to its accumulation at *MAT*, HsRAD52-FLAG displayed limited accumulation at *HML* that was only significantly over background (p = 0.0017) at six hours (**Figure 6C**). This peak, 2.2-fold level of accumulation of HsRAD52-FLAG at *HML* was 5.8-fold lower than the peak accumulation of ScRad52-FLAG, indicative of substantially more restricted association. The accumulation of the HsRAD52-G59R-FLAG and HsRAD52-S346X-FLAG mutant proteins at *HML* were not significantly above background (p ≥ 0.18) at any time point, and were significantly below that of HsRAD52-FLAG at its six hour peak of accumulation (p ≤ 0.05). These results are consistent with both HsRAD52-G59R-FLAG and HsRAD52-S346X-FLAG possessing defects in the ability to associate with *HML* during MTI.

### The *HsRAD52-G59R* and *HsRAD52-S346X* alleles do not support repair synthesis in *rad52* mutant cells

**Figure 7:**
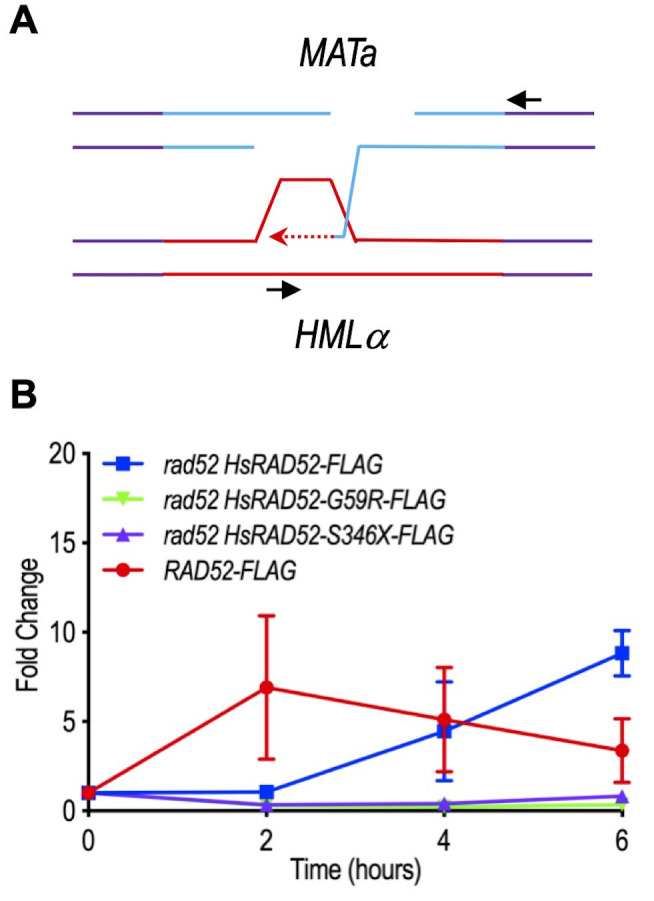
FIGURE 7: The *adh1::HsRAD52-G59R-FLAG* and *adh1::HsRAD52-S346X-FLAG* alleles confer defects in repair synthesis during MTI. **(A)** Cartoon depicting extension by repair DNA synthesis of putative heteroduplex formed upon association between sequences at the *MAT* and *HML* loci during MTI. Following DSB formation at the *MAT* locus (blue lines) exonucleolytic processing creates ssDNA onto which ScRad52-FLAG and HsRAD52-FLAG are proposed to bind. A search for homology putatively results in formation of a heteroduplex linking homologous sequences at the broken *MAT* locus with sequences at the intact *HML* locus (solid red lines). Extension of the heteroduplex by DNA repair synthesis (dotted red line) copies “α” information from *HML* that replaces “a” information lost from *MAT*, ultimately resulting in a change from *MATa* to *MATα.* Repair synthesis ultimately covalently joins “α” information from *HML* with sequences downstream from the *MAT* locus, which can be detected by PCR using the depicted primers (black arrows). **(B)** Repair synthesis was defective in *rad52 adh1::HsRAD52-G59R-FLAG* and *rad52 adh1::HsRAD52-S346X-FLAG* mutant cells. Genomic DNA was collected before immunoprecipitation from the same wild-type (ABX3961-4C), *rad52 adh1::HsRAD52-FLAG* (ABX3977-10C), *rad52 adh1::HsRAD52-G59R-FLAG* (ABX3985-55B), and *rad52 adh1::HsRAD52-S346X-FLAG* (ABX3994-18D) cultures used for the ChIP analyses described above (Fig 6). Extension by repair synthesis of the putative *MAT/HML* heteroduplex was quantitated by semi-quantitative end-point PCR using the primers depicted in panel A. DNA from the intact *SAM1* locus was also quantitated as a signal for normalization. PCR products were separated on agarose gels, stained with ethidium bromide and band intensities were quantified using ImageJ. Normalized mean ratios and corresponding standard deviations from three independent time courses were calculated by dividing signal obtained from repair synthesis with the signal from the *SAM1* control. All time point values were then normalized to the signal obtained before DSB formation (T=0 hrs) and plotted against elapsed time after DSB formation.

The prevailing model for MTI proposes that following DSB formation at the *MAT* locus, *MAT* sequences invade intact, homologous sequences at the *HML* locus forming a heteroduplex that is extended by repair synthesis, which replaces information lost from *MAT* with information from *HML* (**Figure 7A**) [39]. Previous studies found that repair synthesis reached near maximum levels by two hours after DSB formation at the *MAT* locus [54]. Similarly, our studies with *RAD52-FLAG* yeast strains revealed a seven-fold level of accumulation of the repair synthesis intermediate at two hours that was not significantly different at subsequent time points (p ≥ 0.27) (**Figure 7B**). These results are consistent with rapid interaction between *MAT* and *HML* genomic sequences following DSB formation at the *MAT* locus.

In marked contrast to the *RAD52-FLAG* yeast strains, the *rad52 adh1::HsRAD52-FLAG* strains displayed slow accumulation of the repair synthesis intermediate after DSB formation at the *MAT* locus, with levels that were not significantly above background at two hours (p = 0.64), and increasing to a peak accumulation of 8.8-fold at six hours (**Figure 7B**). These data indicate that ScRad52-FLAG supports a more rapid interaction between *MAT* and *HML* genomic sequences, and extension of the heteroduplex by repair synthesis than HsRAD52-FLAG. Importantly, the repair synthesis intermediate did not accumulate significantly (p ≥ 0.16) throughout the time course in either the *rad52 adh1::HsRAD52-G59R-FLAG* or *rad52 adh1::HsRAD52-S346X-FLAG* mutant strains, consistent with HsRAD52-G59R and HsRAD52-S346X failing to support interaction between *MAT* and *HML* genomic sequences, and/or extension of the heteroduplex by repair synthesis. These results are consistent with the differential abilities of the *adh1::HsRAD52-FLAG, adh1::HsRAD52-G59R-FLAG* and *adh1::HsRAD52-S346X-FLAG* alleles to support MTI in *rad52* mutant budding yeast cells (**Figure 3B**; Table S4).

## DISCUSSION

The structure [51, 55–57] and several biochemical activities [58–61] of the RAD52 protein have been conserved from bacteriophage to humans. However, its cellular function has changed substantially through phylogenesis; it plays an integral role in the canonical HRR apparatus in budding yeast [62–64], but a separate role in mammals [19–21]. In support of this diversification, we previously demonstrated that HsRAD52 possesses the distinct ability to promote repair of DSBs by conservative HRR in budding yeast independently from the canonical HRR apparatus [29], which may be similar to its function in mammalian cells [20, 22, 65].

The current study demonstrates that expression of *adh1::HsRAD52-FLAG* in *rad52* mutant budding yeast cells substantially suppressed the X-ray sensitivity (**Figure 2**; Table S4) and MTI defects (**Figure 3**; Table S4). The 70-fold suppression of the *rad52* MTI defect by *adh1::HsRAD52-FLAG* is of particular interest as it demonstrated that HsRAD52-FLAG can function effectively within a highly specialized, heterologous system for DSB repair [66, 67], even though it does not associate with the endogenous HRR machinery [29]. The current studies also indicate that HsRAD52-FLAG and ScRad52-FLAG interacted similarly with recipient sequences at the *MAT* locus (**Figure 6B**; Table S4), but distinctly with donor sequences at the *HML* locus (**Figure 6C**), and that HsRAD52-FLAG supported a slower progression to the repair synthesis step of MTI (**Figure 7B**; Table S4). These results extend our genetic and molecular analyses of HsRAD52 function in conservative HRR in budding yeast, and further confirm that HsRAD52-dependent HRR proceeds through steps and intermediates that are similar to those of ScRad52-dependent HRR, but with distinct kinetics and as part of a distinct apparatus.

In contrast to its incomplete suppression of the MTI defect in *rad52* mutant cells, expression of *adh1::HsRAD52-FLAG* fully complemented the SSA defect (**Figure 4B**; Table S4), suggesting that HsRAD52-FLAG plays distinct roles in the repair of DSBs by HRR and SSA in budding yeast cells. The capacity of HsRAD52-FLAG to replace ScRad52 in SSA is consistent with previous studies that mapped the SSA function of ScRad52 to its N-terminus [29, 44], the area of greatest shared amino acid sequence homology with HsRAD52 (Figure S2). Consequently, the relationship between the primary structure of this region of HsRAD52 and its ability to support SSA in budding yeast will be a subject of continued investigation. Additionally, the capacity of HsRAD52-FLAG to act as a replacement for ScRad52 in SSA suggests that it works in concert with other factors important for SSA in yeast [68–76], which will also be investigated in the future.

Several naturally occurring variants of human HRR genes have been shown to confer defects in HRR and cancer susceptibility [77–79], linking HRR to disease resistance. Accordingly, we investigated the effects on DSB repair in budding yeast of two *HsRAD52* variants identified in African-American women with breast cancer, *HsRAD52-G59R* and *HsRAD52-S346X*; the latter recently reported to attenuate the pathogenicity of *BRCA2* mutations in breast cancer [30]. The *HsRAD52-G59R* variant confers a change in an N-terminal amino acid of HsRAD52 that is conserved between the human and yeast homologs (Figure S2), while *HsRAD52-S346X* deletes 72 amino acids from the C-terminus of HsRAD52, a region that shares little sequence similarity with the yeast homolog. Interestingly, despite their affecting either conserved or divergent regions of the primary sequence of HsRAD52, the variants exerted nearly identical effects on DSB repair at the genetic and molecular levels in budding yeast. Both mutations essentially ablated the capacity of HsRAD52 to support the repair of X ray- and HO endonuclease-generated DSBs by conservative HRR (**Figures 2** and **3**; Table S4). In striking contrast, neither mutation had a significant effect on the repair of a HO-catalyzed DSB by SSA (**Figure 4**; Table S4), establishing that the *HsRAD52-G59R* and *HsRAD52-S346X* are separation-of-function mutations. This indicates that HsRAD52-FLAG supports DSB repair by conservative HRR and non-conservative SSA through distinct mechanisms in budding yeast. Interestingly, expression of the *HsRAD52-S346X* allele in *RAD52*^*-/-*^ mutant mouse embryonic stem cells was recently shown to support a two-fold lower level of DSB repair by SSA than the *HsRAD52* allele [30], suggesting that the function of HsRAD52 in SSA in yeast and mouse cells might be different. However, this reduced level of SSA correlated with reduced nuclear localization of HsRAD52-S346X, which is the likely outcome of loss of the nuclear localization signal from the C-terminus [80], indicating that the reduction in SSA may not be due to any loss of biochemical activity.

We previously employed ChIP to study the effects of HR gene mutations on HR protein function during DSB repair in budding yeast [81], and have used this approach to examine the effects of the *HsRAD52-G59R-FLAG* and *HsRAD52-S346X-FLAG* alleles on the association of HsRAD52-FLAG with the *MAT* and *HML* loci during MTI. Following DSB formation at the *MAT* locus, the HsRAD52-FLAG, HsRAD52-G59R-FLAG and HsRAD52-S346X-FLAG proteins associated with the *MAT* locus with similar kinetics. However, the peak level of accumulation of HsRAD52-G59R-FLAG was two-fold lower than that of HsRAD52-FLAG (p = 0.002) (**Figure 6B**; Table S4). This reduction might result from the change of a small, uncharged glycine at one edge of the putative ssDNA binding cleft in HsRAD52 to a bulky, positively charged arginine [51], which could impede free passage of negatively charged ssDNA into the binding cleft. The extent to which the reduced association of HsRAD52-G59R-FLAG with the DSB at *MAT* contributes to the reduced frequencies of MTI in *rad52 adh1::HsRAD52-G595R-FLAG* cells is unclear (**Figure 3**; Table S4). However, it is interesting to note that a reduction in association of HsRAD52-G59R-FLAG with a DSB might be expected to impact repair by SSA, however no significant effect was observed (p = 0.23) (**Figure 4**; Table S4). This indicates that even the reduced capacity of HsRAD52-G59R-FLAG to associate with DSBs is sufficient for essentially wild-type levels of repair by SSA. This is particularly interesting as the peak level of association of ScRad52-FLAG with the DSB at *MAT* was five-fold higher than that of HsRAD52-FLAG (p < 0.0001) (**Figure 6**), suggesting that levels of association of ScRad52-FLAG and HsRAD52-FLAG with DSBs are well in excess of those necessary for efficient repair by SSA in budding yeast.

Our examination of the association of ScRad52-FLAG with the *MAT* and *HML* loci revealed that it accumulated more slowly at *HML* (**Figure 6B** and **C**), which was previously interpreted as evidence of sequential association with *MAT* and *HML* during MTI [53, 54]. ScRad52-FLAG ultimately reached a substantial 12.6-fold peak level of accumulation at *HML* consistent with frequencies of MTI of near unity in wild-type yeast cells (**Figure 3B**; Table S4). Like ScRad52-FLAG, HsRAD52-FLAG accumulated rapidly at *MAT* after DSB formation, but accumulated much more slowly and to much lower peak levels at *HML.* This could be due to: a) limitations in the sensitivity of the qPCR method used to detect this association in *rad52 adh1::HsRAD52-FLAG* cells where frequencies of MTI were 10-fold lower than in wild-type cells (**Figure 3B**; Table S4); or b) indicative of HsRAD52-FLAG-dependent MTI utilizing the sequences at *HML* in a distinct manner at the molecular level. The differential association of HsRAD52 with the *MAT* and *HML* loci is consistent with its three-order-of-magnitude greater affinity for ssDNA than dsDNA [82]. Importantly, like ScRad52-FLAG, HsRAD52-FLAG supported the extension by repair synthesis of heteroduplex most likely formed as part of a ternary complex with the *MAT* and *HML* loci (**Figure 7**). This indicates that whatever the nature of association between HsRAD52-FLAG and *HML*, repair of the DSB at *MAT* during MTI in *rad52 adh1::HsRAD52-FLAG* cells involved the use of *HML* sequences as the donor template. Alternative hypotheses for how HsRAD52 may support such heteroduplex formation, including increasing the flexibility of dsDNA prior to intercalation with ssDNA [82], transient unpairing of dsDNA in advance of base-pairing with ssDNA [83], or formation of a dynamic, sliding heteroduplex [84] will be investigated further.

We observed that the impact of the *adh1::HsRAD52-G59R-FLAG* and *adh1::HsRAD52-S346X-FLAG* alleles on association of HsRAD52-FLAG with the *MAT* locus during MTI was modest or undetectable (**Figure 6B**). However, both alleles resulted in the failure of HsRAD52-FLAG to associate with the *HML* locus (**Figure 6C**) and failure to extend *MAT-HML* heteroduplex by repair synthesis (**Figure 7**) in *rad52* mutant cells. This is consistent with the observation that X-ray resistance and frequencies of MTI in *rad52 adh1::HsRAD52-G59R-FLAG* and *rad52 adh1::HsRAD52-S346X-FLAG* mutant cells were not significantly different from those of *rad52* mutant cells (**Figures 2** and **3B**; Table S4). This also suggests that these cells are blocked at a step in the repair of DSBs by conservative HRR that occurs between association of HsRAD52-FLAG with ssDNA sequences adjacent to the DSB, and the interaction of the broken sequences with intact, homologous donor sequences in the genome. Intriguingly, the glycine at position 59 in the primary amino acid sequence of HsRAD52 lays at the apex of a loop that extends to the outer periphery of the “domed cap” observed in the crystal structure formed by a multimer of HsRAD52 N-terminal half peptides, an area thought to be involved in binding to dsDNA [51, 56]. This suggests that changing the glycine to an arginine mildly disrupts interaction with ssDNA adjacent to the DSB, but completely blocks interaction with intact double-stranded donor sequences and any subsequent steps in HRR. The potential impact on the behavior of HsRAD52 of deleting 72 amino acids from the C-terminus is more difficult to contemplate as this region has not been crystallized and contains few recognizable motifs. However, the deletion led to the formation of various HsRAD52 multimers in contrast to the single, heptameric form reported previously for the full-length wild-type protein (**Figure 5**) [85]. Previous research showed that the C-terminal half of HsRAD52 can direct self-association separately from the phylogenetically conserved N-terminal self-association domain [48, 86], suggesting that deletion of the C-terminal 72 amino acids may disrupt this signal and affect multimerization. The impact of both structural changes on the capacity of HsRAD52 to associate with dsDNA will be a focus of future investigations.

The observation that *HsRAD52-S346X* both protects against breast cancer in carriers of pathogenic *BRCA2* mutations [30], and confers a loss of HsRAD52-dependent DSB repair by HRR in budding yeast cells suggests that a similar DNA repair defect may contribute to the synthetic lethality thought to promote the protective effect in humans. Interestingly, *HsRAD52-S346X* also confers a defect in DSB repair by SSA in mammalian cells, suggesting that this variant may precipitate multiple DSB repair defects that impinge upon the survivorship of BRCA2-defective cells [30]. Additionally, HsRAD52 has recently been shown to support other HR-dependent functions in mammalian cells [84, 87] that might also support the survival of BRCA2-defective cells, and whose diminution by *HsRAD52-S346X* could negatively affect viability. Further study of the effects of *HsRAD52-S346X* on these functions in human cells should clarify how HsRAD52 contributes to the survival of BRCA2-defective cells.

The current study suggests that our model system for examining HsRAD52 function in budding yeast cells can be used to further explore the relationship between the structure of HsRAD52 and its function in DSB repair by HRR. This novel approach may unlock a diversity of strategies for selectively killing HRR-defective cancers [21]. The utility of this system in defining defects conferred by *HsRAD52* loss-of-function mutations at the molecular level indicates that it could prove useful for examining the mechanism of action of small molecule inhibitors of HsRAD52 at the molecular level in living cells. Inhibitors of HsRAD52-dependent MTI might be potent and selective inhibitors of HRR-deficient cancers by themselves or in combination with other therapeutic strategies, such as the use of inhibitors of poly (ADP ribose) polymerase [88] or ionizing radiation. The growing significance of the HRR-defective phenotype in a broad array of cancers [89–92] suggests that such inhibitors could play an expanding role in future cancer treatment regimens.

## MATERIALS AND METHODS

### Yeast strains

All strains used (Table S1) were isogenic and constructed, maintained and grown using established procedures [93].

### Plasmids

Construction and amplification of plasmids (Table S2) utilized established techniques [94]. The plasmids utilized in the yeast two-hybrid analysis were constructed using pGBT9, a vector containing a *TRP1* selectable marker and sequences that encode the DNA binding domain of the yeast Gal4 protein, and pGAD424, which contains a *LEU2* selectable marker and sequences encoding the transcription activation domain of Gal4 (Clontech, Mountain View, CA, USA). The plasmids pGBT9-HsRAD52 and pGAD424-HsRAD52 express fusions of the Gal4 DNA binding domain and the Gal4 transcriptional activation domain to the N-terminus of wild-type HsRAD52, respectively, and were described in a previous study [29]. Plasmids pGBT9-HsRAD52-G59R and pGBT9-HsRAD52-S346X were constructed from pGBT9-HsRAD52 and express fusions of the mutant HsRAD52-G59R and HsRAD52-S346X proteins to the Gal4 DNA binding domain. Plasmids pLAY700 and pLAY701 were constructed from pGAD424-HsRAD52 and express fusions of HsRAD52-G59R and HsRAD52-S346X to the Gal4 transcriptional activation domain. The plasmids used to express wild-type and mutant HsRAD52 proteins for native complex size determination were constructed using pET28b, which contains a kanamycin resistance marker and sequences for the bacterial expression of proteins tagged at the N-terminus with six, tandem histidine residues (Novagen/Merck, Darmstadt, GDR). The plasmids pLAY855, pLAY970 and pLAY971 were constructed from pET28b and were used to express N-terminally 6-His-tagged wild-type HsRAD52, and mutant HsRAD52-S346X and HsRAD52-G59R respectively.

### Cellular protein detection

Wild-type and mutant HsRAD52-FLAG proteins were detected using Western blot analysis as described previously [29]. Whole cell extracts of cells of the appropriate genotype were prepared by glass bead disruption, and the proteins separated on acrylamide gels before transfer to nylon membranes (Imobilon-P PVDF, Millipore Sigma, St. Louis, MO, US). Proteins were detected with anti-FLAG M2 (Millipore Sigma) and anti-GAPDH (Aviva Systems Biology, San Diego, CA, USA) primary antibodies, goat anti-mouse HRP-conjugated secondary antibody (Thermo Fisher Scientific, Waltham, MA, USA), chemiluminescent signal generation (SuperSignal West Femto Maximum Sensitivity Substrate, Thermo Fisher Scientific), and visualization on X-ray film.

### Determining levels of ionizing radiation resistance

A minimum of 10 five milliliter YPD (1% yeast extract, 2% bacto peptone, 2% dextrose) cultures were inoculated with single colonies of selected yeast strains, grown to a density of 1 – 3 x 10^6^ cells/ml, washed, resuspended in 5 ml of distilled water, and cells counted by hemacytometer. Specified doses of ionizing radiation from a Xrad320 Xray irradiator (Precision X-Ray, North Branford, CT, USA) were applied to aliquots of cell suspension before appropriate dilutions were plated onto YPD medium, and the number of colonies counted after incubation at 30°C for three days. Percent viability was determined by dividing the number of colonies by the number of cells plated and multiplying by 100. 95% confidence intervals and t-test values were calculated with Prism (GraphPad, San Diego, CA, USA).

### Mating type interconversion (MTI) frequency determination

A minimum of 10 one milliliter YPGL (1% yeast extract, 2% bacto peptone, 3% glycerol, 3% lactate) cultures were inoculated with single colonies of selected *MAT****a*** yeast strains with a galactose-inducible HO endonuclease gene inserted into the *TRP1* locus (*trp1::GAL-HO-kanMX*) with or without a deletion of the *HMR* locus (*hmr::hygMX*), and grown overnight at 30°C to a density of 1 – 2 x 10^7^ cells/ml before addition of 20% galactose to a final concentration of 2%. After an additional 1 hour of incubation at 30°C appropriate dilutions were plated onto YPD and incubated at 30°C for three days. The resulting colonies were counted and replica plated onto fresh YPD plates, printed with a lawn of a *MAT****a*** tester strain (R113a) and incubated overnight at 30°C. Incubated prints were replica plated onto synthetic dextrose minimal medium (SD) and incubated overnight at 30°C. Colonies derived from cells that had successfully converted to *MATα* by repair of the HO endonuclease induced DSB at the *MAT* locus mated with the *MAT****a*** tester lawn cells to form prototrophic diploids that showed up as patches of growing cells on SD replicas. Patches that overlapped with colonies on the original YPD plates were counted as recombinants. MTI frequencies were determined by dividing the number of recombinants by the total number of colonies on the original YPD plates. Mean MTI frequencies, 95% confidence intervals and t-test values were calculated with Prism. A select number of putative diploids from each plating were replica plated to sporulation medium and tetrad formation was scored by microscopic examination, demonstrating the presence of functional *MAT****a*** and *MATα* alleles, and indicating successful mating type interconversion (i.e. *MAT****a*** to *MATα*) in the cells generating the original colonies.

### DSB-stimulated ectopic gene conversion (EGC) frequency determination

The frequency of repair of a HO endonuclease catalyzed DSB at the *HIS3* locus on chromosome XV (*his3-*Δ*3'-HOcs*) by conservative homologous recombination with a *his3-*′*MscI* allele proximal to the *LEU2* locus on chromosome III was determined as described previously [29]. A minimum of 10 one milliliter YPGL cultures were inoculated with single colonies of selected strains and grown at 30°C overnight to a density of 1 – 2 x 10^7^ cells/ml before addition of 20% galactose to a final concentration of 2% followed by an additional four hour incubation at 30°C. Dilutions were plated onto YPD medium to assess viability and onto synthetic complete medium lacking histidine (SC-His) to select for recombinants. Plates were incubated at 30°C for three days and the number of colonies counted. Frequencies of EGC were determined by dividing the number of His^+^ recombinants by the number of viable cells plated. Mean EGC frequencies, 95% confidence intervals and t-test values were calculated with Prism. Gene conversion events at the *HIS3* locus in select His^+^ recombinants were verified by Southern blot analysis.

### Assaying recombination between non-tandem direct repeats

We determined the frequency of repair of a HO-catalyzed DSB by non-conservative HR between 3'- and 5'-truncated copies of the *HIS3* coding sequence flanking the *URA3*-marked plasmid YIp5 inserted into the *HIS3* locus on chromosome XV [95]. Single colonies of select strains grown on SC medium lacking uracil (SC-Ura) were used to inoculate a minimum of 10 one milliliter YPGL cultures and grown overnight to a density of 1 – 2x10^7^ cells/ml. Addition of 20% galactose to a final concentration of 2% with additional four hour incubation at 30°C facilitated expression of HO endonuclease from *trp1::GAL-HO-kanMX* and DSB formation at the HO cut site immediately distal to the 3' truncated copy of *his3* (**Figure 4A**). Dilutions were plated onto YPD to determine viability and onto SC-His to select for recombinants. Colonies were counted after incubation at 30°C for three days. Frequencies of DSB-stimulated direct repeat recombination (DSB-DRR) were determined by dividing the number of His^+^ recombinant colonies by the number of viable cells plated. Mean DRR frequencies, 95% confidence intervals and t-test values were calculated using Prism. Select His^+^ recombinants were scored for deletion events by replica plating to SC-Ura and/or Southern blot analysis.

We used a previously described assay [95] to determine rates of spontaneous DRR (S-DRR). We used strains with the identical 3'- and 5'-truncated *his3* repeats flanking the *URA3*-marked plasmid YIp5 inserted into the *HIS3* locus used above, but lacking an HO cut site adjacent to the 3'-truncated repeat (Figure S3A). Single colonies of select strains grown on SC-Ura were used to inoculate a minimum of 10 one milliliter YPD cultures and grown overnight to saturation at 30°C before dilutions were plated onto YPD to assess viability and SC-His to select for recombinants. Colonies were counted after incubation at 30°C for three days and rates of DRR determined by the method of the median [96] as described previously [97].

### Quantitating protein interaction by yeast two-hybrid analysis

Interaction between wild-type or mutant HsRAD52 proteins were quantitated as previously described [29]. The plasmids pGBT9 and pGAD424 and their derivatives (Table S2) were transformed into the yeast strain Y187 (Clontech, Mountain View, CA, USA) and single transformant colonies used to inoculate a minimum of 10 five milliliter culture of SC lacking leucine and tryptophan and grown to saturation at 30°C. A standard protocol was used to prepare cell extracts and determine specific activities of β-galactosidase in Miller units. Mean specific activities, 95% confidence intervals and T-test values were calculated with Prism.

### Examining multimerization of HsRAD52 by gel filtration analysis

Plasmids were constructed using the vector pET28b (Novagen) for expression in bacterial cells of HsRAD52_1-212_ (pLAY855), HsRad52-G59R_(1-212)_ (pLAY971) or HsRAD52-S346X (pLAY970). Plasmids were transformed into Artic Express PR competent *E. coli* cells (Agilent, Santa Clara, CA, USA) with selection on LB medium containing kanamycin at a final concentration of 50 μg/mL. Transformants were used to inoculate liquid LB-kanamycin cultures and grown to the appropriate density (OD_600_ = 0.7) before the induction of protein expression by addition of IPTG to a final concentration of 0.4 mM. Induced cells were grown at 15°C for 20 hours. Cell pellets were harvested by centrifugation, weighed and resuspended in 5 mL of 20 mM Tris HCl, 300 mM NaCl, 20 mM imidazole and 2 mM β-mercaptoethanol (BME), pH 8.0 buffer per gram of cells. Cells were lysed by sonication, cleared by centrifugation for 30 min at 30,000 x g and supernatants loaded onto Ni-NTA affinity columns (HisTrap FF; GE Health Sciences, Westborough, MA, USA). Protein bound to the column was eluted with a linear imidazole gradient (700 mM). Eluted protein was either dialyzed, or fast desalted using a Hi Prep 26/10 Desalting column (GE Health Services), into 20 mM Tris HCl, 50 mM NaCl, 5% glycerol, 2mM BME, pH 8.0. Soluble protein was then applied to a HiPrep Heparin FF 16/10 column (GE Health Sciences) and eluted with a NaCl gradient (700 mM). Fractions containing wild-type or mutant HsRAD52 were run through a size exclusion column (Superdex 200; GE Health Sciences) pre-equilibrated in 10 mM Tris HCl, 300 mM NaCl, 2 mM BME, pH 8.0 at 0.5 mL/min flow rate.

### Determining levels of interaction of ScRad52 and HsRAD52 with donor and recipient loci during MTI

Association of C-terminally FLAG-tagged ScRad52, or wild-type or mutant HsRAD52 with the donor *HML*, recipient *MAT* and control *SAM1* loci during MTI were examined using chromatin immunoprecipitation (ChIP) as described previously [29]. Selected strains were grown in YPGL cultures and aliquots collected before and at various times after addition of galactose to a final concentration of 2%. Cells were collected and extracts prepared before chromatin fragments associated with ScRad52-FLAG or HsRAD52-FLAG were immunoprecipitated, bound to protein A/G beads, and DNA purified from eluted chromatin fragments. The amounts of specific DNA sequences adjacent to the *MAT****a***, *HML* and *SAM1* loci among the immunoprecipitated DNA were determined by quantitative polymerase chain reaction (qPCR) using specific primer sets (Table S3). The *MAT****a*** primer set amplifies a 110 bp sequence 500 bp upstream of the HO cut site in *MAT****a*** on chromosome III. The *HML* primer set amplifies a 187 bp sequence 67 bp downstream from the HO cut site sequence in *HML* on chromosome III. The *SAM1* primer set amplifies a 105 bp sequence 187 bp upstream from the *SAM1* coding sequence on chromosome XII. Each qPCR determination was performed in triplicate on a BioRad CFX96 Touch Real-Time PCR Detection System (BioRad, Hercules, CA, USA) using the following cycling conditions: Step 1, 95°C, 10 minutes; Step 2, 95°C, 30 seconds; Step 3, 56°C, 30 seconds; Step 4, 72°C, 30 seconds; Step 5, Detection; return to step 2 and repeat 39 times; Step 6, melt curve analysis from 65°C to 95°C. Data were analyzed using the Bio-Rad CFX Manager 3.1. Cycle quantification (Cq) values were reported in triplicate and outliers, defined as those outside a range of 0.5 cycles were excluded from calculations. No template controls yielded Cq values outside the acceptable range (>35). Association of ScRad52-FLAG, or wild-type and mutant HsRAD52-FLAG with the donor and recipient sequences was recorded as fold enrichment determined by degree of occupancy at the *HML* and *MAT****a*** loci relative to occupancy at the *SAM1* reference locus and relative to degree of occupancy before addition of galactose to the cultures (t = 0), and calculated by the Livak method [98]. Mean fold changes in degree of occupancy and standard deviations were calculated from at least 10 technical replicates drawn from at least three independent biological replicates. T-test values were calculated with Prism.

### Determining levels of repair synthesis during MTI

Repair of the DSB at *MAT****a*** using sequences from *HML* as template during MTI was followed over time using semi-quantitative PCR with DNA sequences recovered from the same chromatin fragments prepared for ChIP analysis, but collected prior to the immunoprecipitation step. The amount of extension by repair synthesis of *MAT* sequences cleaved by HO-endonuclease using the intact *HML* locus as template was determined by PCR using the SIext-f and SIext-r primers (Table S3) as described previously [54]. These primers were chosen as they are complementary to genomic sequences unique to *MAT* and *HML* and lay as close as possible to the sequences required for MTI. This strategy results in an amplicon that is 500 bp in length and too long for quantitation using qPCR. The following cycling conditions were used: Step 1, 95°C, 2 minutes; Step 2, 95°C, 20 seconds; Step 3, 64°C, 10 seconds; Step 4, 70°C, 10 seconds; Step 5, return to step 2 and repeat 34 times; Step 6, 70°C, 5 minutes. The same primers for amplifying sequences adjacent to *SAM1* described above were used to generate signals for normalization using the following cycling conditions: Step 1, 95°C, 10 minutes; Step 2, 95°C, 30 seconds; Step 3, 56°C, 30 seconds; Step 4, 72°C, 30 seconds; Step 5, return to step 2 and repeat 29 times; Step 6, 72°C, 5 minutes. PCR products were separated by electrophoresis on 1.5% agarose gels, stained with ethidium bromide (10µg/ml), and quantitated using ImageJ (imagej.net). Fold changes in normalized levels of repair synthesis were reported as fold differences from levels observed before addition of galactose to the growth medium (t = 0). Mean fold changes and standard errors were calculated from at least three independent biological replicates using Prism.

## SUPPLEMENTAL MATERIAL

Click here for supplemental data file.

All supplemental data for this article are available online at www.microbialcell.com/researcharticles/2020a-clear-microbial-cell/.

## Abbreviations:

DRR: – direct repeat recombination;
DSB: – double-strand break;
HsRAD52: – human RAD52;
HR: – homologous recombination;
HRR: – HR repair;
IR: – ionizing radiation;
MTI: – mating type interconversion;
NHEJ: – non-homologous end joining;
ScRAD52: – yeast RAD52.
