# Supplementary material for: Variants of the human RAD52 gene confer defects in ionizing radiation resistance and homologous recombination repair in budding yeast
Source: Microb Cell. 2020 Jul 20;7(10):270–85. doi: 10.15698/mic2020.10.732 (PMC7517009; doi:10.15698/mic2020.10.732)
Supplement: Supplementary file 1 [file mic-07-270-s01.pdf]

SUPPLEMENTAL MATERIAL

Table S1 – *Saccharomyces cerevisiae* strains used in this study

STRAIN	GENOTYPE [a]	ASSAY	ORIGIN
ABM325	*MAT*__a__*::LEU2 his3-Δ3'-HOcs-URA3-his3-Δ5' trp1::GAL-HO-kanMX*	DSB-DRR [b]	This study
ABM326	*MAT*__a__*::LEU2 his3-Δ3'-HOcs-URA3-his3-Δ5' trp1::GAL-HO-kanMX rad52::TRP1*	DSB-DRR	This study
ABM507	*MAT*__a__*::LEU2 his3-Δ3'-HOcs-URA3-his3-Δ5' trp1::GAL-HO-kanMX rad52::TRP1 adh1::HsRAD52-hygMX-ADH1*	DSB-DRR	This study
ABX3479-1A	*MATα his3-Δ3'-URA3-his3-Δ5'*	S-DRR [c]	This study
ABX3479-2C	*MAT*__a__ *his3-Δ3'-URA3-his3-Δ5' rad52::TRP1*	S-DRR	This study
ABX3479-3A	*MAT*__a__ *his3-Δ3'-URA3-his3-Δ5' rad52::TRP1 adh1::HsRAD52-hygMX-ADH1*	S-DRR	This study
ABX3566	*MAT*__a__*/α HIS3/his3-11, 15 URA3/ura3-1*	IRR [d]	Manthey et al., 2017
ABX3568	*MAT*__a__*/α HIS3/his3-11, 15 URA3/ura3-1 rad52::TRP1/rad52::TRP1*	IRR	Manthey et al., 2017
ABX3666-37B	*MAT*__a__*::LEU2 his3-Δ3'-HOcs LEU2-his3-ΔMsc* I *trp1::GAL-HO-kanMX*	DSB-EGC [e]	Manthey et al., 2017
ABX3684-12B	*MATα LEU2 adh1::HsRAD52-FLAG-hygMX-ADH1*	Western [f]	This study
ABX3697-82D	*MAT*__a__*::LEU2 his3-Δ3'-HOcs LEU2-his3-ΔMsc* I *trp1::GAL-HO-kanMX rad52::TRP1*	DSB-EGC	Manthey et al., 2017
ABX3720-6C	*MAT*__a__*::LEU2 his3-Δ3'-HOcs LEU2-his3-ΔMsc* I *trp1::GAL-HO-kanMX rad52::TRP1 adh1::HsRAD52-G59R-FLAG-kanMX-hygMX-ADH1*	DSB-EGC	This study
ABX3782-2D	*MATα URA3 adh1::HsRAD52-G59R-FLAG-kanMX-hygMX-ADH1*	Western	This study
ABX3817-6C	*MAT*__a__ *ADE2 trp1::GAL-HO-kanMX rad52::TRP1 adh1::HsRAD52-hygMX-ADH1*	MTI [g]	This study
ABX3817-7D	*MAT*__a__ *ADE2 trp1::GAL-HO-kanMX rad52::TRP1*	MTI	This study
ABX3817-15B	*MAT*__a__ *ADE2 trp1::GAL-HO-kanMX*	MTI	This study
ABX3885-17B	*MAT*__a__*::LEU2 his3-Δ3'-HOcs LEU2-his3-ΔMsc* I *trp1::GAL-HO-kanMX rad52::TRP1 adh1::HsRAD52-FLAG-kanMX-hygMX-ADH1*	DSB EGC	Manthey et al., 2017
ABX3933-46C	*MAT*__a__ *hmr::hygMX trp1::GAL-HO-kanMX*	MTI	This study
ABX3943-3B	*MAT*__a__ *hmr::hygMX trp1::GAL-HO-kanMX rad52::TRP1*	MTI<br>ChIP [h]	This study

ABX3943-7A	*MAT**a** hmr::hygMX trp1::GAL-HO-kanMX rad52::TRP1 adh1::HsRAD52-FLAG-kanMX-hygMX-ADH1*	MTI<br>ChIP<br>RS [i]	This study
ABX3961-4C	*MAT**a** hmr::hygMX trp1::GAL-HO-kanMX URA3 RAD52-FLAG-kanMX*	MTI<br>ChIP<br>RS	This study
ABX3970-88A	*MAT**a**::LEU2 his3-Δ3'-HOcs-URA3-his3-Δ5' trp1::GAL-HO-kanMX rad52::TRP1 adh1::HsRAD52-G59R-FLAG-kanMX-hygMX-ADH1*	DSB-DRR	This study
ABX3974-11C	*MAT**a** ADE2 adh1::HsRAD52-S346X-FLAG-kanMX-hygMX-ADH1*	Western	This study
ABX3975-15A	*MAT**a**::LEU2 his3-Δ3'-HOcs-URA3-his3-Δ5' trp1::GAL-HO-kanMX rad52::TRP1 adh1::HsRAD52-S346X-FLAG-kanMX-hygMX-ADH1*	DSB-DRR	This study
ABX-3976-2A	*MAT**a**::LEU2 his3-Δ3'-HOcs LEU2-his3-ΔMsc* I *trp1::GAL-HO-kanMX rad52::TRP1 adh1::HsRAD52-S346X-FLAG-kanMX-hygMX-ADH1*	DSB-EGC	This study
ABX3985-55B	*MAT**a** HIS3 hmr::hygMX trp1::GAL-HO-kanMX rad52::TRP1 adh1::HsRAD52-G59R-FLAG-kanMX-hygMX-ADH1*	MTI<br>ChIP<br>RS	This study
ABX3994-18D	*MAT**a** hmr::hygMX trp1::GAL-HO-kanMX rad52::TRP1 adh1::HsRAD52-S346X-hygMX-ADH1*	MTI<br>ChIP<br>RS	This study
ABX4129	*MAT**a**/α HIS3/his3-11, 15 URA3/ura3-1 rad52::TRP1/rad52::TRP1 adh1::HsRAD52-G59R-FLAGkanMX--hygMX-ADH1/adh1::HsRAD52-G59R-FLAG-kanMX-hygMX-ADH1*	IRR	This study
ABX4130	*MAT**a**/α HIS3/his3-11, 15 URA3/ura3-1 rad52::TRP1/rad52::TRP1 adh1::HsRAD52-FLAG-kanMX-hygMX-ADH1/adh1::HsRAD52-FLAG-kanMX-hygMX-ADH1*	IRR	This study
ABX4131	*MAT**a**/α HIS3/his3-11, 15 URA3/ura3-1 rad52::TRP1/rad52::TRP1 adh1::HsRAD52-S346X-FLAG-kanMX-hygMX-ADH1/adh1::HsRAD52-S346X-FLAG-kanMX-hygMX-ADH1*	IRR	This study
R113a	*MAT**a** ADE2 CAN1 HIS3 LEU2 TRP1 URA3 tyr1*	MTI tester	Rodney Rothstein

[**a**] All strains are isogenic and possess the following genotype unless otherwise noted: *ade2-1 can1-100 his3-11, -15 leu2-3, -112 trp1-1 ura3-1*
[**b**] Double-strand break stimulated direct repeat recombination
[**c**] Spontaneous direct repeat recombination
[**d**] Ionizing radiation resistance
[**e**] Double-strand break stimulated ectopic gene conversion

[f] Western blot
[g] Mating type interconversion
[h] Chromatin immuno-precipitation
[i] Repair synthesis

Table S2 – Plasmids used in this study

NAME	DESCRIPTION	SOURCE
pGBT9	Multi-copy bacterial-yeast shuttle vector containing a *TRP1* selectable marker and the coding sequence of Gal4 DNA binding domain	Clontech
pGBT9-HsRAD52	Derivative of pGBT9 for expression of wild-type HsRAD52-Gal4 DNA binding domain fusion	Manthey et al., 2017
pGBT9-HsRAD52-G59R	Derivative of pGBT9-HsRAD52 for expression of mutant HsRAD52-G59R-Gal4 DNA binding domain fusion	This study
pGBT9-HsRAD52-S346X	Derivative of pGBT9-HsRAD52 for expression of mutant HsRAD52-S346X-Gal4 DNA binding domain fusion	This study
pGAD424	Multi-copy bacterial-yeast shuttle vector containing a *LEU2* selectable marker and the coding sequence of Gal4 transcription activation domain	Clontech
pGAD424-HsRAD52	Derivative of pGAD424 for expression of wild-type HsRAD52-Gal4 transcription activation domain fusion	Manthey et al., 2017
pLAY700	Derivative of pGAD424-HsRAD52 for expression of mutant HsRAD52-G59R-Gal4 transcription activation domain fusion	This study
pLAY701	Derivative of pGAD424-HsRAD52 for expression of mutant HsRAD52-S346X-Gal4 transcription activation domain fusion	This study
pET28b	Plasmid containing *kanR* selectable marker for expression of N-terminally 6-His-tagged proteins in *E. coli*	Novagen
pLAY855	pET28b expressing N-terminally 6-His-tagged wild-type $HsRAD52_{(1-212)}$	This study
pLAY970	pET28b expressing N-terminally 6-His-tagged mutant HsRAD52-S346X	This study
pLAY971	pET28b expressing N-terminally 6-His-tagged mutant $HsRAD52\text{-}G59R_{(1-212)}$	This study

Table S3 – Oligonucleotide primers used in this study

NAME	SEQUENCE	APPLICATION
MATYaF-1812	5' – CAA TAT CAC CCC AAG CAC G – 3'	*MAT**a*** recipient ChIP
MATYaR-1922	5' – GAA GTG GAG TAA TGC CAC – 3'	*MAT**a*** recipient ChIP
HMLz2F-3852	5' – CAG ACT CAA GCA AAC AAT C – 3'	*HML*α donor ChIP
HMLR-4040	5' – GAG GGC ACA AGG AAC ACG – 3'	*HML*α donor ChIP
SAMF-(-192)	5' - CAC TCT GGT AGC GAT GAA A – 3'	*SAM1* reference for ChIP and repair synthesis
SAMR-(-106)	5' - CGA TGA ATA ACA GAC AAC AC – 3'	*SAM1* reference for ChIP and repair synthesis
SIext-f	5' - GCA GCA CGG AAT ATG GGA CT – 3'	Repair synthesis
SIext-r	5 – ATG TGA ACC GCA TGG GCA GT – 3'	Repair synthesis

Table S4 – Master Data

Genotype	IRR [(a)]	DSB-DRR [(b)]	S-DRR [(c)]	MTI [(d)]	DSB-EGC [(e)]
WT	33.2 (30.0, 36.5)[(f)] [1]	$2.27x10^{-1}$ (1.82, 2.72) [1]	$3.86x10^{-5}$ [1]	$5.96x10^{-1}$ (5.53, 6.40) [1]	$1.72x10^{-3}$ [(g)] (1.37, 2.07) [1]
rad52Δ	$4.3x10^{-2}$ (2.2, 6.3) [-767] [(h)]	$3.41x10^{-2}$ (2.18, 4.65) [-6.7]	$1.94x10^{-6}$ [-19.9]	$8.17x10^{-4}$ (5.81, 10.53) [-729]	$9.21x10^{-7}$ [(g)] (6.52, 11.90) [-1868]
rad52Δ HsRAD52	n.d. [(i)]	$2.26x10^{-1}$ (1.50, 3.01) [+1.0]	$7.10x10^{-5}$ [+1.8]	$6.49x10^{-2}$ (4.53, 8.45) [-9.2]	$7.20x10^{-5}$ [(g)] (5.73, 8.68) [-23.9]
rad52Δ HsRAD52-FLAG	$8.8x10^{-1}$ (7.1, 10.4) [-37.7]	n.d.	n.d.	$5.66x10^{-2}$ (3.07, 8.25) [-10.5]	$3.96x10^{-5}$ [(g)] (1.13, 6.79) [-43]
rad52Δ HsRAD52-G59R-FLAG	$2.9x10^{-2}$ (1.7, 4.1) [-1145]	$1.74x10^{-1}$ (1.25, 2.24) [-1.3]	n.d.	$1.17x10^{-3}$ (0.58, 2.07) [-509]	$2.67x10^{-6}$ (1.80, 3.55) [-644]
rad52Δ HsRAD52-S346X-FLAG	$4.7x10^{-2}$ (2.6, 6.7) [-706]	$3.82x10^{-1}$ (2.18, 5.46) [+1.7]	n.d.	$1.74x10^{-3}$ (0.62, 3.11) [-343]	$1.16x10^{-6}$ (0.50, 1.83) [-1483]

[(a)] Ionizing Radiation Resistance (% viable cells after exposure to 320 Gy of X-ray radiation)
[(b)] Double-strand Break Stimulated Direct Repeat Recombination Frequency (recombinants/viable cell)
[(c)] Spontaneous Direct Repeat Recombination Rate (events/cell/generation)
[(d)] Mating Type Interconversion Frequency (recombinants/viable cell)
[(e)] Double-strand Break Stimulated Ectopic Gene Conversion Frequency (recombinants/viable cell)
[(f)] 95% confidence interval
[(g)] From Manthey et al, 2017
[(h)] Fold difference from wild-type
[(i)] Not Determined

Table S5 – Y2H Data

Plasmids	ß-Galactosidase Activity (Miller Units)
pGAD424/pGBT9	0.010<br>(0.008, 0.012) [a]<br>[1]
pGAD424/pGBT9-HsRAD52	0.008<br>(0.005, 0.011)<br>[-1.3] [b]
pGAD424-HsRAD52/pGBT9	0.012<br>(0.006, 0.017)<br>[+1.2]
pGAD424-HsRAD52/<br>pGBT9-HsRAD52	0.795<br>(0.642, 0.948)<br>[+80]
pGAD424-HsRAD52-G59R/<br>pGBT9-HsRAD52-G59R	1.039<br>(0.773,1.306)<br>[+104]
pGAD424-HsRAD52-S346X/<br>pGBT9-HsRAD52-S346X	0.878<br>(0.646, 1.111)<br>[+88]

[a] 95% confidence interval
[b] Fold difference from wild-type

Figure S1

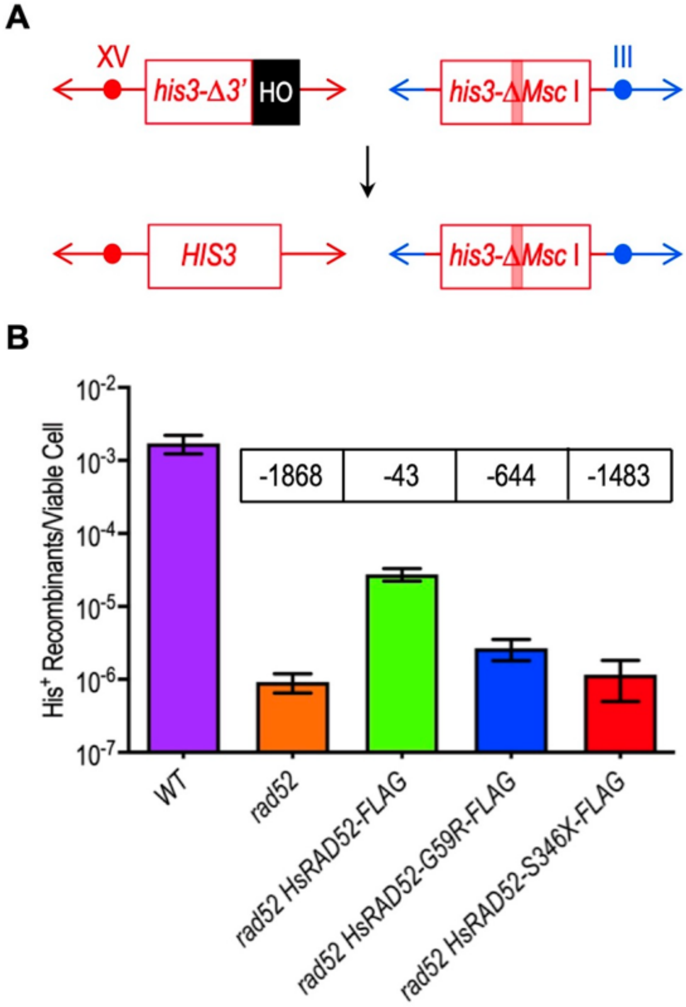

<u>The *adh1::HsRAD52-G59R-FLAG* and *adh1::HsRAD52-S346X-FLAG* alleles do not suppress the ectopic gene conversion defect of *rad52* mutant yeast cells</u>

A. <u>Cartoon depicting DSB-stimulated *his3* ectopic gene conversion (EGC) assay</u> The *his3-Δ3'-HOcs* substrate (white "*his3Δ-3'* box) at the *HIS3* locus on chromosome XV (red double ended arrow) substitutes a 127 bp DNA fragment containing an HO cut site (black "HO" box) for 238 bp of the 3' end of the *HIS3* coding sequence and flanking DNA. The *his3-ΔMsc* I substrate (white "*his3-ΔMsc* I" box) proximal to the *LEU2* locus on chromosome III (blue double ended arrow) is comprised of a 1.8 kb genomic clone containing the *HIS3* gene that has been disrupted by the insertion of a 10 bp *Not* I linker into the *Msc* I site in the coding sequence (pink bar). Repair of a HO-catalyzed DSB at the *his3-Δ3'-HOcs* substrate by unidirectional transfer of information from the *his3-ΔMsc* I substrate (black arrow) creates an intact *HIS3* gene.

B. <u>The *adh1::HsRAD52-G59R-FLAG* and *adh1::HsRAD52-S346X-FLAG* alleles confer defects in DSB-stimulated EGC</u> Single colonies of haploid wild-type (ABX3666-37B), *rad52* (ABX3697-82D), *rad52 adh1::HsRAD52-FLAG* (ABX3885-17B), *rad52 adh1::HsRAD52-G59R-FLAG* (ABX3720-6C), and *rad52 adh1::HsRAD52-S346X* (ABX3976-2A) strains carrying the EGC assay components were used to inoculate at least

10 one milliliter YPGL cultures and grown overnight. Following a period of expression of HO endonuclease, appropriate dilutions were plated onto YPD medium to determine viability or medium lacking histidine to select for recombinants and incubated for three days at 30°. Colonies were counted and frequencies of EGC determined by dividing the number of His^+ recombinants by the number of viable cells plated. Mean frequencies of EGC and 95% confidence intervals were plotted against genotype. Fold differences below (-) wild-type are in boxes above the plot of each mean frequency.

Figure S2

```
                                                20                                             40                                             60
ScRAD52   MN----EIMD MDEKKPVFGN HS-------- ------EDIQ TKLDKKLGPE YISKRVGFGT  42
HsRAD52   MSGTEEAILG GRDSHPAAGG GSVLCFGQCQ YTAEEYQAIQ KALRQRLGPE YISSRMAGGG  60
Consensus M*GTEE*I** *****P**G* *SVLCFGQCQ YTAEEY**IQ **L***LGPE YIS*R***G*
                                                80                                            100                                            120
ScRAD52   SRIAYIEGWR VINLANQIFG YNGWSTEVKS VVIDFLDERQ GKFSIGCTAI VRVTLTSGTY 102
HsRAD52   QKVCYIEGHR VINLANEMFG YNGWAHSITQ QNVDFVDLNN GKFYVGVCAF VRVQLKDGSY 120
Consensus ****YIEG*R VINLAN**FG YNGW****** ***DF*D*** GKF**G**A* VRV*L**G*Y
                                               140                                            160                                            180
ScRAD52   REDIGYGTVE NERRKPAAFE RAKKSAVTDA LKRSLRGFGN ALGNCLYDKD FLAKIDKVKF 162
HsRAD52   HEDVGYGVSE GLKSKALSLE KARKEAVTDG LKRALRSFGN ALGNCILDKD YLRSLNKLPR 180
Consensus *ED*GYG**E ****K****E *A*K*AVTD* LKR*LR*FGN ALGNC**DKD *L****K***
                                               200                                            220                                            240
ScRAD52   DPP-DFDENN LFRPTDEIS- ESSRTNTLHE NQEQQQYPNK RRQLTKVTNT NPDSTKNLVK 220
HsRAD52   QLPLEVDLTK AKRQDLEPSV EEARYNSCRP NMALG-HP-- --QLQQVTS- -PSRPSHAV- 232
Consensus **PL**D*** **R***E*SV E**R*N**** N****Q*PNK RRQL**VT*T NP******VK
                                               260                                            280                                            300
ScRAD52   IENTVSRGTP MMAAPAEANS KNSSNKDTDL KSLDASKQDQ DDLLDDSLMF SDDFQDDDLI 280
HsRAD52   ---------- -IPADQDCSS RSLSSSAVES EATHQRKLRQ KQL------- QQQFRE---- 270
Consensus IENTVSRGTP M**A*****S ***S****** ******K**Q **LLDDSLMF ***F**DDLI
                                               320                                            340                                            360
ScRAD52   NMGNTNSNVL TTEKDPVVAK QSPTASSNPE AEQITFVTAK AATSVQNERY IGEESIFDPK 340
HsRAD52   RMEKQQVRVS TPS-----AE KSEAAPPAPP VTHSTPVTVS ---------- ---EPLLEKD 312
Consensus *M******V* T**KDPVVA* *S**A***P* ****T*VT** AATSVQNERY IGEE******
                                               380                                            400                                            420
ScRAD52   YQA---QSIR HTVDQTTSKH IPASVLKDKT MTTARDSVYE KFAPKGKQLS MKNNDKELGP 397
HsRAD52   FLAGVTQELI KTLEDNSEKW ---AVTPD-- ---AGDGVVK ---PSSRADP AQTSDTL--- 358
Consensus **AGVTQ*** *T******K* IPA*V**DKT MTTA*D*V** KFAP****** ****D**LGP
                                               440                                            460                                            480
ScRAD52   HMLEGAGNQV PRETTPIKTN ATAFPPAAAP RFAPPSKVVH PNGNGAVPAV P-QQRSTRRE 456
HsRAD52   ----ALNNQM ------VTQN RT---PHSVC HQKPQAK--- -SGSWDLQTY SADQRTTGNW 401
Consensus HMLE***NQ* PRETTP***N *TAFPP**** ***P**KVVH P*G******* *A*QR*T***

ScRAD52   VGRPKINPLH ARK--PT 471
HsRAD52   ESHRKSQDMK KRKYDPS 418
Consensus ****K***** *RKYDP*
```

Alignment of primary amino acid sequences of yeast and human RAD52 proteins The amino acid sequences of Rad52 and HsRAD52 were aligned using the CLC Sequence Viewer (CLC Bio, Aarhus, Denmark). Identical residues are highlighted in blue.

Figure S3

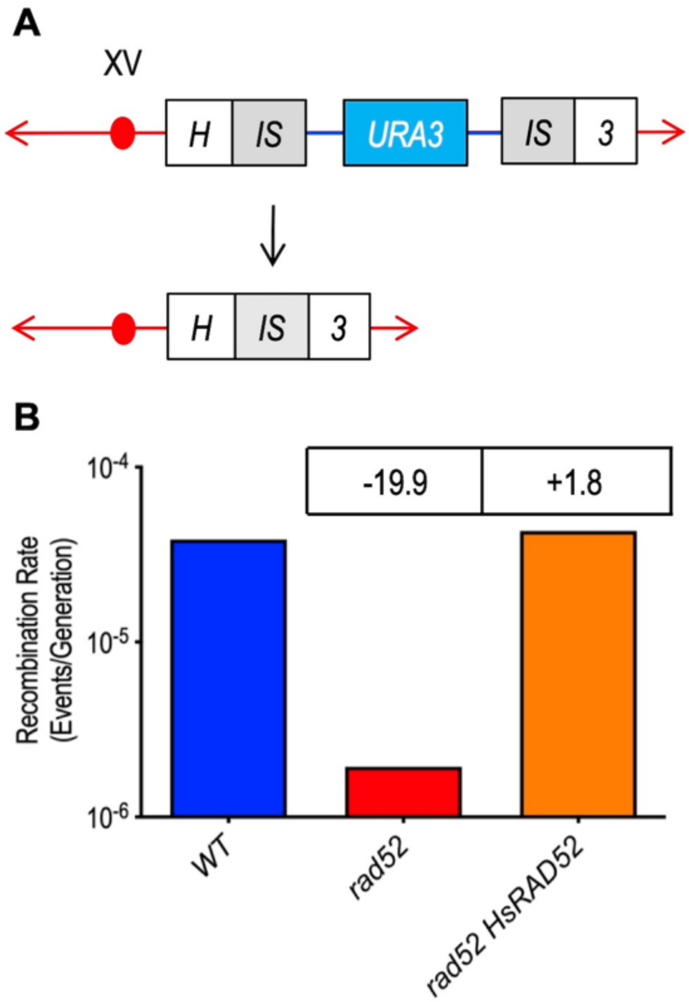

<u>The *adh1::HsRAD52* allele complements the loss of spontaneous direct repeat recombination in *rad52* mutant yeast cells</u>

A. <u>Cartoon depicting assay for spontaneous recombination between non-tandem direct repeats</u> Spontaneous recombination between duplicate segments of the *HIS3* coding sequence (gray *IS* boxes) flanking a 5kb *URA3*-marked (aqua box) plasmid (blue bar) inserted into the *HIS3* locus on chromosome XV creates an intact *HIS3* gene while deleting the plasmid.

B. <u>The *adh1::HsRAD52* allele supports wild-type rates of spontaneous DRR in *rad52* mutant cells</u> Single colonies of haploid wild-type (ABX3479-1A), *rad52* (ABX3479-2C), and *rad52 adh1::HsRAD52-FLAG* (ABX3479-3A) strains were used to inoculate a minimum of 10 one milliliter cultures of synthetic medium lacking uracil and grown overnight. Appropriate dilutions were plated onto solid YPD medium to determine viability, and onto medium lacking histidine to select for recombinants. Following incubation for three days at 30° colonies were counted and frequencies of DRR determined by dividing the number of His^+ recombinants by the number of viable cells plated. Rates of DRR were plotted against genotype. Fold differences above (+) and below (-) wild-type are in boxes above the plot of each mean frequency.

Figure S4

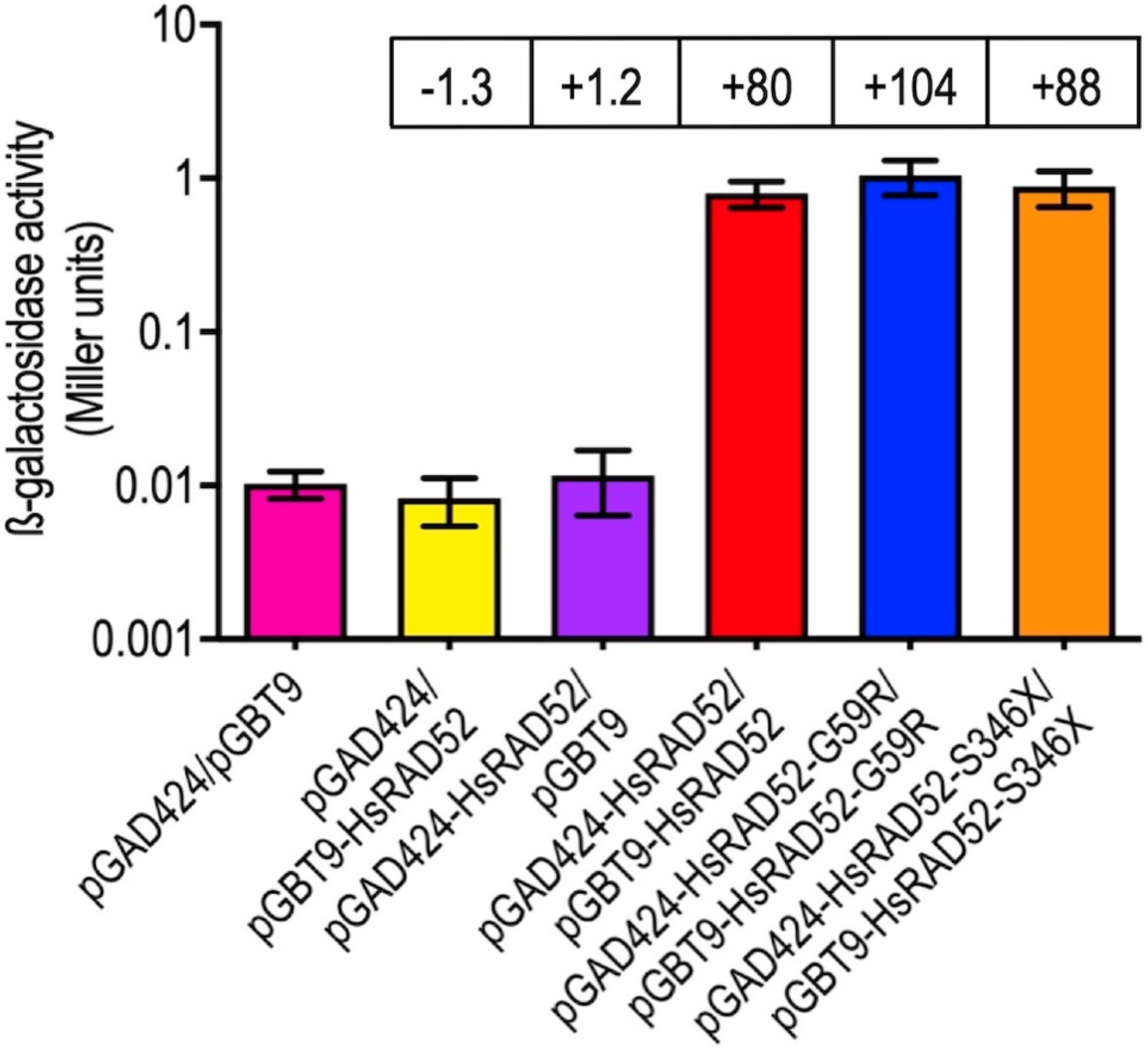

HsRAD52-G59R and HsRAD52-S346X display wild-type levels of self-association in the yeast two-hybrid assay Single colonies of the yeast strains bearing plasmids containing genes encoding N-terminal fusions of wild-type or mutant HsRAD52 to the transcriptional activation and DNA binding domains of Gal4 were used to inoculate at least 10 five milliliter cultures of synthetic medium lacking leucine and tryptophan and grown to saturation at 30°C. Fold differences above (+) and below (-) background (pGAD424/pGBT9) mean ß-galactosidase activity levels are in boxes above the plot.
